# Vertebrate TNF Superfamily: Evolution and Functional Insights

**DOI:** 10.3390/biology14010054

**Published:** 2025-01-10

**Authors:** Ignacio Marín

**Affiliations:** Instituto de Biomedicina de Valencia, Consejo Superior de Investigaciones Científicas (IBV-CSIC), 46010 Valencia, Spain; imarin@ibv.csic.es

**Keywords:** TNF, tumor necrosis factor, whole-genome duplication, network, ligand evolution, cell signaling, adaptive immunity

## Abstract

This study characterizes the evolution of the tumor necrosis factor superfamily (TNFSF) across vertebrate lineages, both cyclostomes and gnathostomes, by combining sequence similarity and synteny data for the genes from 23 model species. The available evidence supports a simple model in which most of the diversity found in living species can be attributed to the expansion of four genes found in an ancestor of all vertebrates before the first of the genome duplications that occurred in the vertebrate lineages. It is inferred that the ancestor of all cyclostomes possessed only six TNFSF genes. A cyclostome-specific genome triplication had little effect on the total number of these genes. The ancestor of all gnathostomes, due to the effect of a second genome duplication plus additional single-gene duplications, already had 21 TNFSF genes. In several gnathostome lineages, particularly in some tetrapods, the TNF superfamily has significantly contracted due to numerous gene losses. This evolutionary model provides a framework for exploring functional data, showing that the descendants of different ancestral genes have acquired distinct roles, most prominently in the innate and adaptive immune systems, which led to a species-specific refinement of which TNFSF genes were conserved or lost. Several data hitherto difficult to interpret (the interactions of very different TNFSF ligands with the same receptors; the ability of the same ligands to bind alternative receptors, with or without death domains; and the cooperation of different ligands in specific functions) can be explained as consequences of the evolutionary history of the TNF superfamily.

## Simple Summary

In animals, the proteins encoded by genes of the tumor necrosis factor superfamily (TNFSF) play crucial roles in cell signaling. They regulate cell differentiation, survival, and programmed cell death in multiple organs and tissues, with particularly significant functions in the immune system. This study characterizes the TNFSF genes present in all major vertebrate lineages and proposes a model for the evolution of this superfamily since the emergence of vertebrates. The model can serve as a tool to interpret functional data and to generate new hypotheses regarding the roles of TNFSF genes in multiple species.

## 1. Introduction

One of the most important cell-signaling systems in animal species involves interactions between ligands of the tumor necrosis factor superfamily (TNFSF) and their receptors, members of the tumor necrosis factor receptor superfamily (TNFRSF; [1,2,3,4,5]). The evolution of the TNF and TNFR superfamilies is still incompletely understood. Several works have explored the patterns of diversification of the TNF superfamily in vertebrates (e.g., [6,7,8,9,10,11,12,13,14]), but only the most recent analyses have included early-diverging vertebrate branches such as chondrichthyans [13,14] and cyclostomes [14]. Despite all these works, we still lack a coherent model of how the TNF superfamily has evolved since the origin of vertebrates. A first hypothesis was proposed by Collette et al. [6], based on the limited dataset of thirty TNFSF genes, from just eight osteichthyan species, available in 2003. In that study, phylogenetic trees, based on sequence similarity, were analyzed together with synteny data, which provides sequence-independent evidence about gene relationships. They proposed that all vertebrate TNFSF genes originated from a single ancestral gene that, after a few tandem duplications and gene losses and a transposition event, produced four genes, arranged as two pairs in separate chromosomes. Then, they suggested that the two rounds of whole-genome duplications (WGDs) that occurred in vertebrates significantly expanded the TNFSF gene repertoire: eight genes in four chromosomes after WGD1 and fifteen genes in eight chromosomes after WGD2. These fifteen genes would correspond to most of those present in extant osteichthyan species, including humans, with only a few lineage-specific losses and duplications occurring subsequently [6]. Although inaccurate in the details, this model can still be considered broadly correct today. The two main features of the Collette et al. model, i.e., that the vertebrate ancestor possessed a limited number of TNFSF genes and that most genes found now in living species emerged in the vertebrate WGDs, explaining both their chromosomal distribution and sequence similarities, have stood the test of time.

In recent years, two studies have proposed updated models for the evolution of the TNF superfamily in vertebrates. In an analysis published four years ago [14], I examined one hundred and sixty-eight sequences from ten model species which spanned the whole vertebrate spectrum (five osteichthyans, two chondrichthyans, and three cyclostomes). Phylogenetic analyses using precise maximum-likelihood methods, combined with the detailed synteny data available for all these species, resulted in a model that, while conceptually similar to that of Collette et al. [6], differed in important details. Only three genes were postulated in the vertebrate ancestor before the WGDs, a single gene on a chromosome and two genes, produced by a tandem duplication, on a second chromosome. Then, after WGD1, a single tandem duplication occurred that brought the total number of genes to seven. After that, the second WGD plus two individual duplications and a gene loss led also, as in the Collette et al. model, to a total of fifteen genes [14]. However, the remodeling of the TNF superfamily has been much more extensive, so much so that it could be deduced from the very limited set of species analyzed in Collette et al. [6]. For instance, I identified eight genes in fishes unknown two decades ago. As a result, only 12 of the 15 hypothesized genes after WGD2 were the same in my model [14] and that of Collette et al. [6]. The inclusion of a broader range of species allowed me to establish for the first time that different gnathostome lineages evolved very differently after WGD2. Some chondrichthyans experienced important gene amplifications, while other lineages suffered duplications and losses of genes in similar numbers. For example, in humans, nine duplications and six deletions since WGD2 were inferred [14].

The third model, proposed by Redmond et al. [13], was based on the analysis of 353 TNFSF sequences from 28 osteichthyan species (although for many species, only one or a few genes were included) and 8 chondrichthyans, using a methodology nearly identical to that in my previous study [14]. However, the model they proposed was notably more complex, i.e., it involved hypothesizing many more ancient genes, than the models just described. They interpreted the data to suggest that nine genes were present in the ancestor of all vertebrates prior to WGD1, eighteen genes were already present after WGD1, and thirty after WGD2, which was, however, followed by the immediate loss of nine genes. It is very significant that, with only one exception which I will describe below, all the genes analyzed by Redmond et al. [13] had already been characterized in my study [14], only that they interpreted some of them as having a much older origin. Therefore, the stark differences between the models proposed in these two studies do not stem from new evidence, but rather from fundamentally different interpretations of the phylogenetic trees and the synteny data, a discrepancy that warrants further study.

In order to settle the question, data from cyclostomes are crucial. Analyzing this early branching group is essential for any study aiming to trace the evolution of a gene family in vertebrates. Furthermore, it is now well established that cyclostomes diverged from the rest of the vertebrates after WGD1 but before WGD2 [15,16,17,18]. Thus, their genomes provide direct information on which genes were already present after the first genomic duplication. However, although cyclostome TNFSF data were available and were even analyzed in my first study [14], Redmond et al. [13] surprisingly did not include any cyclostome species in their more recent study, despite the obvious fact that analyzing those species allowed for a direct test of their model. In particular, they hypothesized 18 TNFSF genes after WGD1, a proposal that was difficult to reconcile with the fact that cyclostomes possess only about half that number, as I had already demonstrated [14]. On the other hand, while I analyzed in depth the evolution of the cyclostome TNFSF genes, my conclusions were marred by the prevailing belief at the time that WGD2 occurred before the cyclostome/gnathostome split [19,20,21]. As a result, I had to conclude that the limited set of TNFSF genes found in cyclostomes was due to a large number of gene losses in the cyclostomata lineage following WGD2, a deduction that, if cyclostomes, as is well established today, did not suffer that duplication, may be mistaken. In summary, none of the published works to date have correctly established the relationships between cyclostome and gnathostome TNFSF genes. Only by resolving this can we expect to be able to develop a general model for the evolution of the TNF superfamily in vertebrates. This is precisely the goal of this study. By expanding and updating the data from my previous work and refining the methods used, I provide a detailed characterization of the evolution of the TNF superfamily across all vertebrate lineages, which leads to a new, comprehensive model for the emergence, expansion and, in some cases, elimination of TNFSF genes in both cyclostomes and gnathostomes. This new model, which is actually simpler than all published before, sheds light on significant functional data, offering new insights into how the complexity of the TNF superfamily impinged on vertebrate development and the diversification of the vertebrate immune system.

## 2. Materials and Methods

***Nomenclature of the TNF superfamily***: To name the genes of this superfamily in gnathostome species, I will use, whenever possible, the standardized names described by Locksley et al. [1] and Aggarwal et al. [4]. Table 1 (next page) shows those names, along with the ones that refer to the same genes in our species and other synonyms used for mammalian genes. In addition to these genes, in my previous study [14], I discovered six additional genes absent in tetrapods, which I named *TNFSF-Fish1* to *TNFSF-Fish6* (abbreviated from now on as *TNFSF-F1* to *-F6*). Later, I will describe that one of them, *TNFSF-F4*, is actually present in tetrapod species and propose a modification of its name. Additional standardized names for some newly detected TNFSF genes in specific gnathostomes, as well as for all those present in cyclostome species, will be detailed in the Section 3. Finally, ancient genes will be named as *TNFSF-Vx*, where “V” stands for vertebrate, indicating that they are pan-vertebrate genes, already present before the cyclostome/gnathostome split, and “x” corresponds to a number, conveniently selected to facilitate tracing their evolutionary relationships.

***Sequence retrieval, alignments, and phylogenetic analyses***: The sequences of the TNFSF genes present in 23 model species spanning the entire spectrum of vertebrate lineages were obtained following the iterative search method already used in my earlier analysis [14]. In brief, TNFSF sequences of these species in the nr, wgs, est, tsa, htgs, and gss databases at the National Center for Biotechnology Information (NCBI; http://ncbi.nlm.nih.gov) were identified through TBLASTN searches, using, as queries first, the protein sequences of the human TNFSF genes, later using the sequences obtained in these human-specific searches in the same way, until no additional TNFSF sequences were detectable. This approach was designed to prevent the loss of divergent sequences with low similarity to the human TNFSF genes. From these sequences, the highly conserved and functionally critical THD domain [5], the only region that can be reliably aligned in all TNFSF sequences, was selected. After eliminating duplicates and partial sequences (i.e., those with less than 90% of their THD domain), the total number of TNFSF genes detected was 385, with 335 belonging to gnathostome species and 50 to cyclostomes.

Given that the previous analyses [13,14], despite having similar datasets, substantially differed in the tree topologies obtained, it was deemed crucial to compare different alternatives to obtain the best possible multiple-sequence alignment and the most reliable tree topology. Here, therefore, all sequences were aligned using 12 different algorithms. Nine of them (FFTNS1, FFTNSF2, NWNS1, NWNS2, FFTNSI, NWNSI, GINSI, LINSI, and EINSI) are included in the MAFFT program (version 7.5.0.5; [22]). The other three were ClustalX (Version 2.1; [23]), Clustal Omega (Version 1.2.0; [24]), and MUSCLE (version 5.1.0; [25]). In all cases, the default settings implemented in the programs were used. To perform FFTNSI, NWNSI, GINSI, LINSI, and EINSI analyses, the user must select a maximum number of cycles of refinement of the alignment, which was set to 1000.

Maximum likelihood (ML) analyses for tree construction were performed using the IQTREE program (version 2.2.0; [26]) in two separated steps. First, the optimal substitution model for each of the 12 alignments was determined using ModelFinder [27]. Then, the ML trees based on the optimal substitution models were obtained. However, several studies have shown that it is essential to use different perturbation strengths (i.e., several values of the *pers* parameter used by IQTREE) and to repeat the tree search multiple times (*runs* parameter > 1) to obtain optimal ML trees for a given alignment. Thus, following the recommendations of the IQTREE developers [28,29], and as implemented in some of my previous works [14,30,31], the following parameters were used: the *pers* parameter was alternatively set at 0.2, 0.5, or 0.8; the number of replicates to stop the analysis (*nstop* parameter) was set to 500; and the number of independent runs (*runs* parameter) was set to 10. The number of ultrafast bootstrap replicates (*bb* parameter; [32,33]) used to assess the reliability of the tree topology was set to 1000. Consequently, a total of 36 different ML trees (from 12 different alignment algorithms, each being analyzed with 3 alternative perturbation strengths) were obtained. In principle, the best out of these 36 alternatives should be the one with the highest ML value. However, since each protein substitution model implies a specific number of degrees of freedom and the alignments varied in length, it is more appropriate to compare the trees using the Bayesian Information Criterion (BIC; [34]), which corrects for these differences. Thus, the optimal ML tree was considered to be the one with the lowest BIC value among the 36 alternatives. Regarding the evaluation of the topology of the tree, branches with bootstrap values of at least 95% are considered to have a significant statistical support [32].

***Synteny determination***: Local synteny data were collected to identify genes adjacent to the ones of the TNF superfamily. This information complements the phylogenetic trees, given that, when putative orthologous TNFSF genes (i.e., genes that the trees suggest are the same in different species, because they have very similar sequences and appear clustered together) are surrounded by the same genes in two or more species, it is most likely that they are true orthologs. In fact, given the large total number of genes in vertebrates, the presence of even a single identical adjacent gene in two species is already strong evidence for orthology (e.g., the probability of this occurring by chance is *p* = 2 × 10^−4^ in a genome with 20,000 genes). Alternatively, if a WGD has occurred, TNFSF genes with identical adjacent genes in two different species may be either orthologs or ohnologs (given that the whole region became duplicated in that WGD), but all of them will still be derived from a single gene. Thus, in both scenarios, with and without a WGD, putative orthologies deduced from the trees can be validated by a type of information, gene location, which is totally independent from the sequence data from which those trees were generated.

Although I previously analyzed the local synteny of the regions containing TNFSF genes in 10 of the 23 species selected for this study [14], all synteny data were for this study repeated from scratch to incorporate any improvements in the genomic sequences that have arisen in recent years. Particularly important are the data from cyclostomes, which were quite incomplete when I compiled them for my previous work. Here, three newly assembled cyclostome genomes have been analyzed [18,35,36], and also data from a substantially improved *Petromyzon marinus* genome assembly [37] and an unpublished *Myxine glutinosa* assembly (GenBank GCA_040869285.1), which has become recently available, have been included. To establish at least two upstream and two downstream protein-coding genes adjacent to the TNFSF genes in all these species (up to four were characterized in some cases, when deemed necessary), different strategies were followed. For species with fully annotated genome assemblies at NCBI, the proteins translated from the deduced genes were used as queries in TBLASTN searches with default parameters, against the human non-redundant protein dataset (NCBI RefSeq_protein database). If multiple splice variants were deduced for a given gene, the largest protein was chosen. The best TBLASTN hits were considered to correspond to the most probable human orthologs, and their positions in the human genome were retrieved. On the other hand, for genomes lacking detailed gene annotations, the determination of the most likely human orthologs was based on BLASTX analyses, again with default parameters, wherein adjacent 100 Kb-long DNA fragments around each TNFSF gene were translated in all six frames and compared with the human proteins in the RefSeq_protein database. Again, the top hits detected in these searches were considered to be the most likely human orthologs, and, for each of them, their positions in the human genome were recorded. I found in my previous study (Supplementary File S2 in [14]), and it will be confirmed here, that congruent results are obtained for both closely and distantly related species, which validates these methods. After finishing all these analyses, I observed that a few genes were missing in three model species (*Gallus gallus*, *Callorhinchus millii*, and *Amblyraja radiata*), which were, however, present in evolutionarily close relatives. To fill this gap in the synteny results, which could hamper the characterization of orthologies in both birds and chondrichthyans, the proteins produced by genes adjacent to some TNFSF genes in the genomes of the birds *Cuculus canorus* and *Rissa tridactyla*, the shark *Carcharodon carcharias*, and the sawfish *Pristis pectinata* were analyzed using the same procedures.

***Modeling the early evolution of the TNF superfamily in vertebrates***: A model integrating sequence and synteny data with the known phylogeny of the analyzed species was developed, following strategies already used in my first analysis [14]. The steps are the following: (1) Tree topology is used to establish orthology groups and also to determine the closeness of those groups in order to characterize sets of closely related paralogs, derived from an ancestral gene either by local duplications or WGDs. (2) Synteny information is used to confirm the orthologies deduced in the previous step and to establish the most likely origin of the regions where the TNFSF genes are located. By analyzing a group of adjacent genes in multiple species, it is generally possible to determine whether they have a common origin, not only in those cases when the regions contain orthologs, but also when they include ohnologs generated in WGDs. (3) The known evolutionary relationships among the model species are used to interpret the similarity of orthologs and paralogs according to parsimony criteria, i.e., out of the different explanations that may fit the data, the one which requires the minimum number of uncommon evolutionary events, such as gene duplications or losses, is preferred.

***Deducing functions for ancient TNFSF genes***: The strategy developed in [14] was applied. Starting with the known functions of the mammalian TNFSF genes, a backward analysis was performed, in such a way that when two close paralogs with known origins (e.g., ohnologs, both derived from a single gene due to a given WGD) have a common functional feature, it was assumed that their common ancestor already had that feature. This assumption, which requires a single emergence for a given functional capability, is the most parsimonious explanation for both paralogs sharing the feature, given that the alternative is that they acquired it twice independently since they started to diverge.

***Expression data***: In order to establish correlations between gene evolutionary dynamics and gene function, expression data for the 18 human TNFSF genes across 81 cell types were retrieved from The Human Protein Atlas, version 23 (https://www.proteinatlas.org/; single cell type section; [38]). Data for each gene are expressed in transcripts per million, i.e., number of transcripts that come from a particular gene out of a million of transcripts characterized. In the first analysis, the correlations in the patterns of expression of all human TNFSF genes were determined. Two genes were deemed significantly correlated if the probability of finding by chance a given value of Spearman’s correlation index for their expression levels in the cell types analyzed was *p* < 5 × 10^−2^, after correcting the data both for rank ties and for multiple tests using the False Discovery Rate (FDR) method [39]. In a second type of analysis, the specific expression similarity of immune system cells was analyzed. First, for each gene, the expression levels in all 81 tissues were ordered from highest to lowest. Then, data for the eleven cell types of that system present in The Human Protein Atlas (macrophages, monocytes, dendritic cells, granulocytes, Langerhans cells, Hofbauer cells, Kupffer cells, T-cells, B-cells, NK-cells, and plasma cells) were analyzed. Two TNFSF genes were considered to have a similar profile in immune cells when, out of those 11 cell types, there were at least 3 in common whose levels of expression were among the top 10 highest values for both genes. The likelihood of observing that congruence by chance has a very low probability, *p* (x ≥ 3) = 5.4 × 10^−4^, according to a binomial distribution. Once the significant pairs of genes were established in these two analyses (global correlation and immune system expression similarity), each gene was considered a node in a network and positive results were traced as links between genes. Gephi (0.10; [40]) was used to visualize the corresponding networks.

## 3. Results

### 3.1. TNF Superfamily Diversity in All Vertebrate Lineages

**Figure 1:**
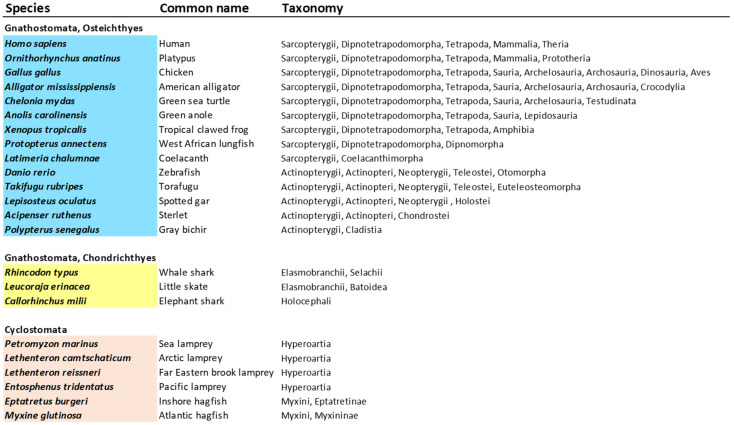
Model species used in this study. Scientific names, common names, and main taxonomic units to which they belong are indicated. Blue: osteichthyans. Yellow: Chondrichthyans. Sand: cyclostomes.

The TNF superfamily genes found in 23 vertebrate model species, 17 gnathostomes, and 6 cyclostomes, selected to represent all the main vertebrate lineages (Figure 1), were characterized.

Regarding gnathostomes, some distinct lineages not included in any previous works, such as batoidea (rays), cladistia (bichirs), and chondrostei (sturgeons), were here incorporated. These analyses include all the sequences, a total of one hundred and forty-seven, detected in nine sarcopterygians, representatives of all the main lineages of that clade. This is a much more complete dataset than those analyzed in previous works. In my first study [14], only forty-three sequences from two sarcopterygian species (*Homo sapiens* and *Latimeria chalumnae*) were studied, while Redmond et al. [13], included 149 sarcopterygian sequences, but from 20 different species, because they did not try to obtain all their TNFSF genes, but just sampled some of them. For the nine species selected in this study, Redmond et al. [13] identified only eighty sequences, which is, according to my data, just 54% of the TNFSF genes that they have. The TNF superfamily genes present in the genomes of four lampreys and two hagfishes were also characterized. In lampreys, seven of them were found in *Entosphenus tridentatus*, nine in *Petromyzon marinus* and *Lethenteron camtschaticum*, and eleven in *Lethenteron reissneri.* In the inshore hagfish (*Eptatretus burgeri*), eight were detected, while only six were found in the other hagfish species analyzed, *Myxine glutinosa*. Thus, the total number of cyclostome sequences obtained was 50, more than doubling those characterized in my previous study [14]. Most new cyclostome sequences were derived from genomes that were then unavailable. However, two *P. marinus* genes and a single *L. camtschaticum* gene were not yet present in the databases at that time and have been included here.

### 3.2. Characterization of Gnathostome Orthogroups Indicates That the TNF Superfamily Is More Complex than Hitherto Known

**Figure 2:**
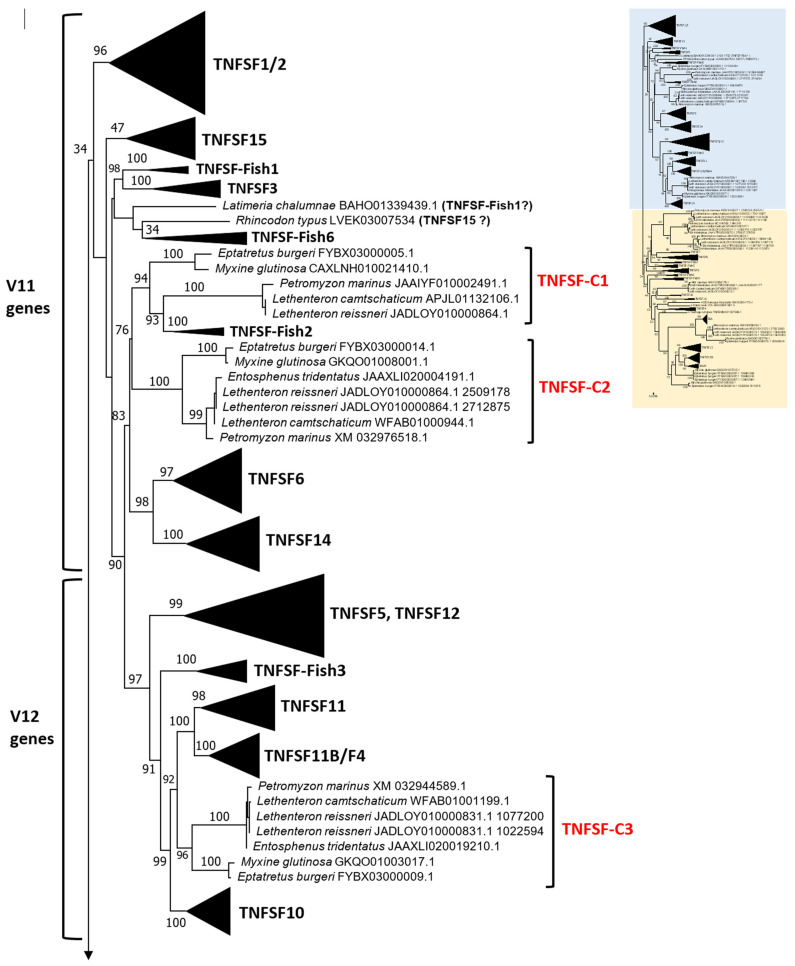

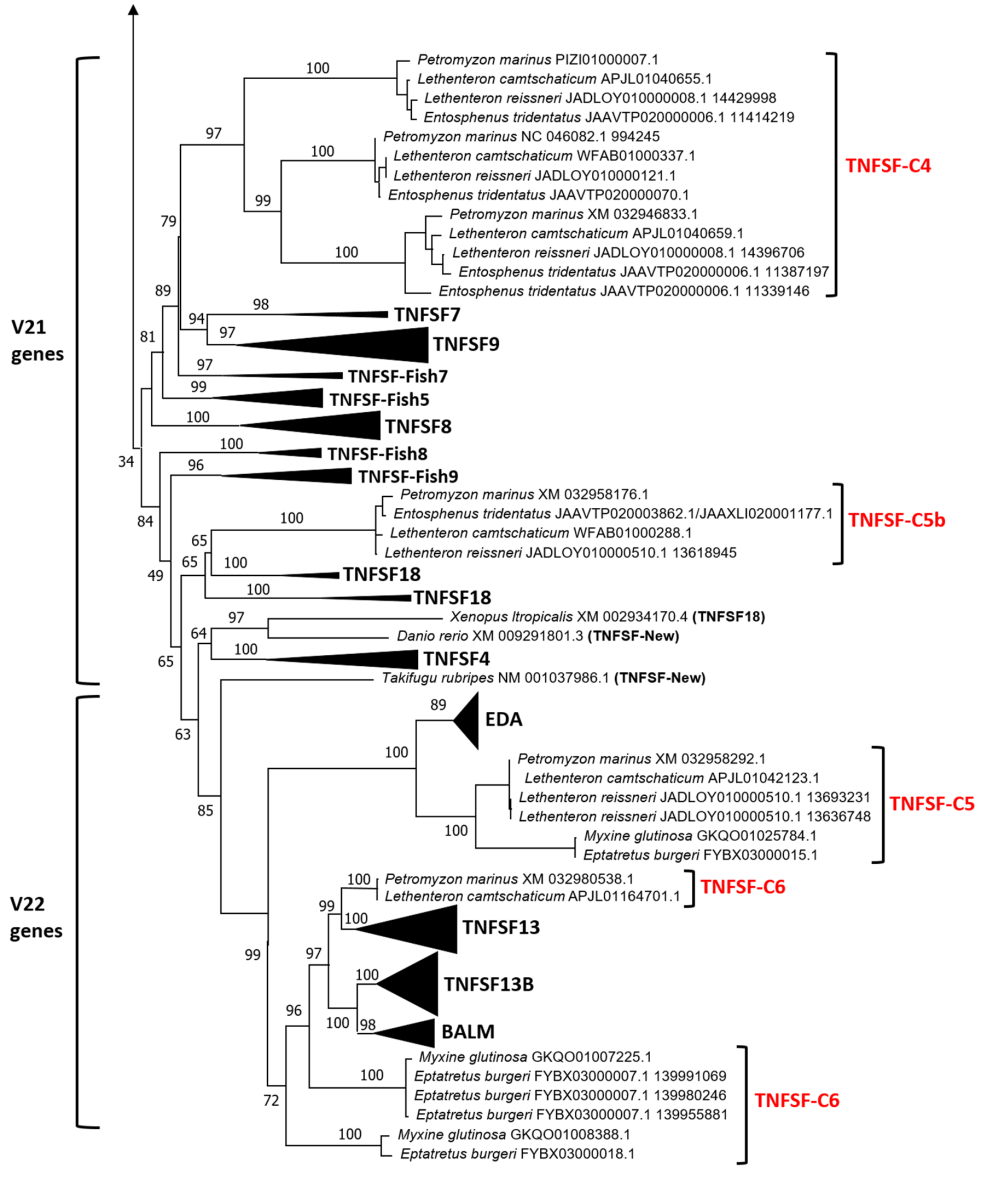
Maximum likelihood tree, orthogroups, and ancestral division of TNFSF genes. The tree is shown in two sections for better visualization. The inset shows each section in different colors. Gnathostome sequences belonging to the same orthogroup have been, when possible, grouped (black triangles). Cyclostome genes (*TNFSF-C1* to *-C6*, in red) are detailed. V11, V12, V21, and V22 refer to regions of the tree derived from four different ancestral genes (see main text). Number refer to bootstrap support (%). The tree was constructed using an alignment produced by the MAFFT-NWNSI algorithm. The optimal ML tree was generated with IQTREE with the following parameters: (1) WAG+F+R5 protein substitution model and (2) perturbation strength = 0.5. The LnL value was −76,863.616.

When the 385 sequences detected in the model species were aligned and analyzed as described in the Section 2, the best tree obtained (both highest ML and lowest BIC) was the one shown in Figure 2. The tree has been divided into two sections to facilitate visualization. The corresponding optimal alignment can be found in Supplementary File S1, and a fully expanded version of the tree (in Newick format) is available in Supplementary File S2. According to both their ML and BIC values, this tree, obtained using the MAFFT-NWNSI algorithm, is much better than those derived for the same sequences with ClustalX, used in my first study [14], or MAFFT-LINSI, which was chosen in Redmond et al. [13].

This type of tree can be dissected at two different levels. First, it is possible to determine orthogroups, that is, groups of orthologous genes present in multiple species, characterized by strong sequence similarity, causing them to appear very close in the tree. Orthologs also often share identical genome locations across species. Such analyses were already performed in the earlier studies, but with quite different results. In my prior analysis [14], 24 orthogroups were characterized in gnathostomes while 23 were identified in Redmond et al. [13]. However, only 17 of these orthogroups are equivalent in both studies, i.e., they include either identical or almost identical sequences in the same groups of species. These were the following: TNFSF1/2, TNFSF5, TNFSF6, TNFSF10, TNFSF11, TNFSF12, TNFSF13, TNFSF13B, TNFSF14, TNFSF15, EDA, BALM, and TNFSF-F1 to -F5. In three other cases, TNFSF3, TNFSF8, and TNFSF18, the groups are not identical, because Redmond et al. [13] identified putative orthologs in sharks that, apparently, I did not detect [14]. They also found a potentially new shark-specific gene, which they named TNFSF33. Finally, two differences are due to Redmond et al. [13] lumping together as orthologous some genes that I considered different: TNFSF4 was merged with TNFSF-New and TNFSF7 with TNFSF9. That these two associations are unsupported by the data will be discussed below. In any case, combining the results of the two studies, and assuming no additional genes remain to be discovered, a maximum of 25 orthogroups would be expected in gnathostomes: the 24 already described in my study [14], plus the additional shark-specific gene identified by Redmond et al. [13].

**Figure 3:**
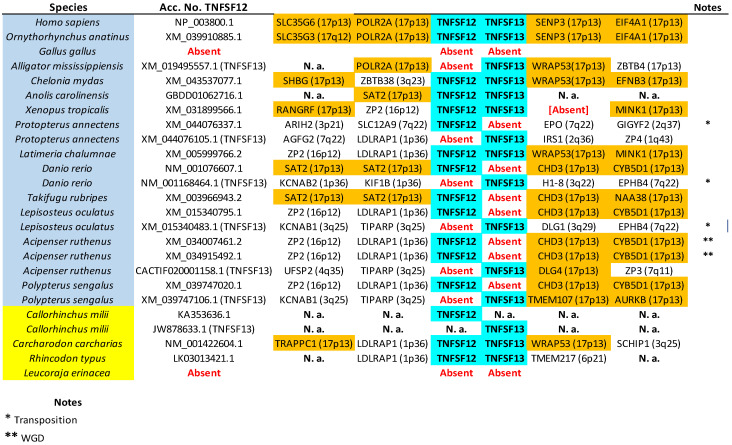
Synteny data for the region surrounding the *TNFSF12* and *TNFSF13* genes in gnathostome species. *Carcharodon carcharias* data have been included due to the lack of genomic information in its close relative *Callorhinchus milii*. Genes are color-coded as follows: (1) blue: TNFSF gene; (2) orange: gene whose most likely ortholog in humans is located on the same chromosome where the analyzed TNFSF genes are found. In this and subsequent figures containing synteny information, “N. a.” indicates that the corresponding region was not available in the databases.

**Figure 4:**
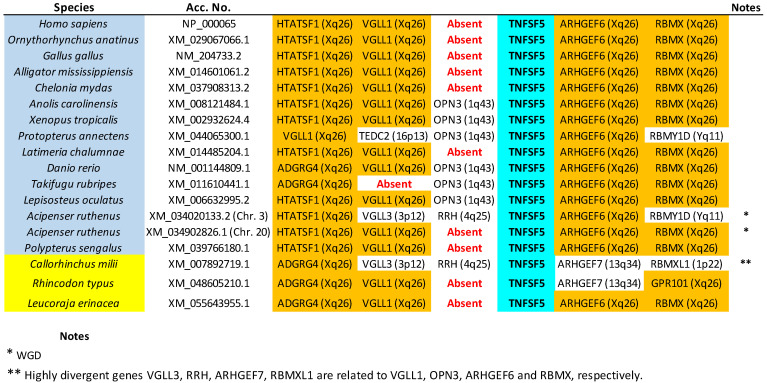
Synteny data for the regions where gnathostome *TNFSF5* genes are located. Color conventions as in Figure 3.

**Figure 5:**
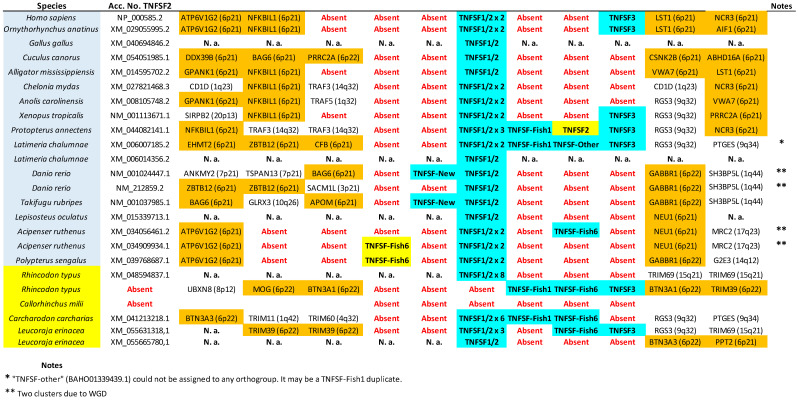
Synteny data for the region surrounding *TNFSF2*, which contains a cluster of TNFSF genes in gnathostomes. *Cuculus canorus* and *Carcharodon carcharias* have been included as substitutes of, respectively, *G. gallus* and *C. milii*, in which data for this region are incomplete. In this and the following figures, genes are color-coded as follows: (1) blue: TNFSF gene; (2) yellow: TNFSF gene in an abnormal position due to transposition or inversion events; (3) orange: gene whose most likely ortholog in humans is located on the same chromosome where the analyzed TNFSF genes are found. In this and subsequent figures containing synteny information, “N. a.” indicates that the corresponding region was not available in the databases.

**Figure 6:**
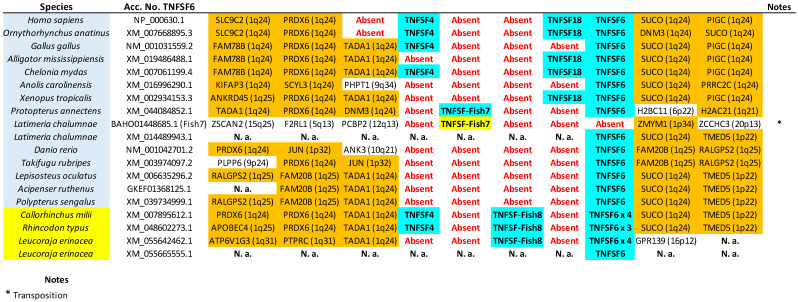
Cluster of TNF superfamily genes around TNFSF6 in the model gnathostome species. In this and subsequent figures containing synteny information, “N. a.” indicates that the corresponding region was not available in the databases.

**Figure 7:**
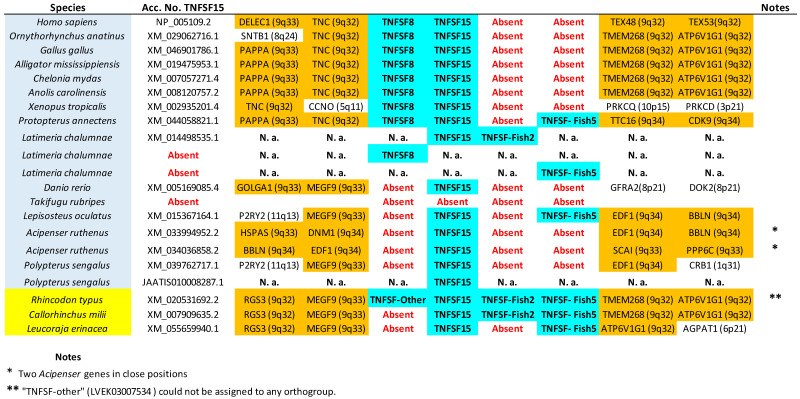
*TNFSF15* is located in tandem with other TNFSF genes in most gnathostome species. In this and subsequent figures containing synteny information, “N. a.” indicates that the corresponding region was not available in the databases.

**Figure 8:**
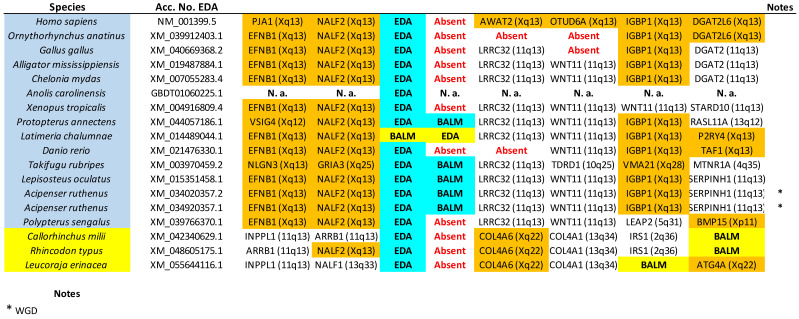
Region where the EDA and BALM genes are located in gnathostomes. Genes are in an inverted orientation in Latimeria. In this and subsequent figures containing synteny information, “N. a.” indicates that the corresponding region was not available in the databases.

**Figure 9:**
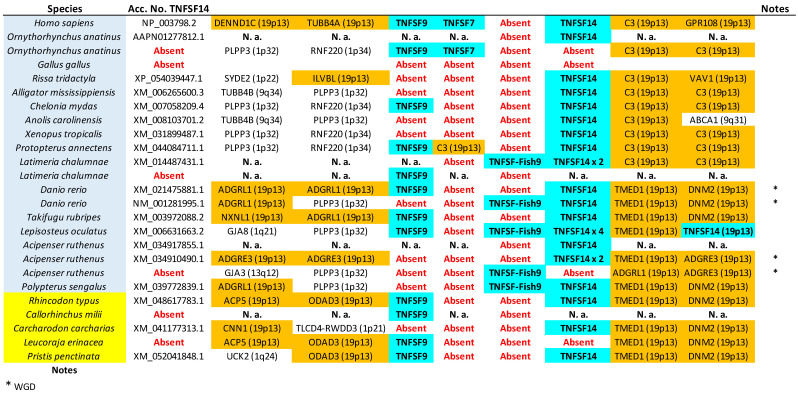
*TNFSF9* and *TNFSF14* are adjacent in both chondrichthyans and osteichthyans. *TNFSF7* and *TNFSF-F9* are located between these two genes in some species. Data from *Rissa tridactyla*, *Carcharodon carcharias*, and *Pristis penctinata* have been included to compensate the fact that their closely related model species (*G. gallus*, *C. milii*, and *L. erinacea*, respectively) apparently do not have some of these genes. In this and subsequent figures containing synteny information, “N. a.” indicates that the corresponding region was not available in the databases.

The tree based on sequence similarity shown in Figure 2, combined with synteny data, (detailed in Figure 3, Figure 4, Figure 5, Figure 6, Figure 7, Figure 8, Figure 9 and Supplementary File S3, which includes the complete synteny results along with additional notes, useful to interpret the results), demonstrates that the gnathostome TNF superfamily is quite more complex than assumed so far. A total of twenty-eight TNFSF orthogroups can be defined in gnathostome species, namely the twenty-four already characterized in my previous work, which this new analysis fully confirms, plus four additional ones. These newly identified groups have been named, given that they are absent in tetrapods and following my previous convention, TNFSF-Fish6 to TNFSF-Fish9 (abbreviated as TNFSF-F6 to -F9). All but two of the three hundred and thirty-five genes characterized in the gnathostome model species can be included in one of these twenty-eight groups. The genes in 21 of these 28 orthogroups are together in branches with significant (≥95%) bootstrap support and also show congruent synteny. The exceptions are TNFSF5, TNFSF12, TNFSF15, TNFSF18, TNFSF-New, TNFSF-F6, and EDA. The first two, TNFSF5 and TNFSF12, fail to appear as two clearly separated branches, because the chondrichthyan *TNFSF5* genes are closer to *TNFSF12* genes than to their orthologs in osteichthyan species (see Supplementary File S2). This was already noted in my previous analysis [14]. However, as already concluded in that study, two different orthology groups can be defined incorporating synteny data. In many species, both in osteichthyans and chondrichthyans, the *TNFSF12* and *TNFSF13* genes are adjacent in a location that in humans corresponds to region 17p13 (Figure 3), while all *TNFSF5* genes, again both in osteichthyans and chondrichthyans, are located in a totally different genomic region in a different chromosome. In humans, it corresponds to Xq26 (Figure 4). The lack of separation in the tree is likely due to different rates of evolution in the *TNFSF5* genes of osteichthyans and chondrichthyans, with the latter either evolving at a slower pace, thereby retaining more similarity to *TNFSF12* genes, or, alternatively, having convergent features with them.

For the TNFSF18 and TNFSF-New orthogroups, the sequences appear in two or even three separated branches, close to one another in the tree (Figure 2). However, it is easy to determine that these related branches include orthologs, as the genes have the same genomic positions in closely related species (Figure 5: *TNFSF-New* genes are present only in *Danio* and *Takifugu*; Figure 6: *TNFSF18* is found in some tetrapods). Finally, all *TNFSF15*, *TNFSF-F6*, or *EDA* gene sequences indeed appear together in the tree, but within branches with non-significant (i.e., <95%) bootstrap support. For *TNFSF15*, this lack of support was already noted before [14], and the reason is that the genes of a few osteichthyan fishes evolve very rapidly, distorting the results (see details in Supplementary File S2). In the other two cases, the most likely explanation is the presence of similar paralogous genes in other species, which may have been included within these groups in some bootstrap replicates, lowering the support values. For *TNFSF-F6*, the closer sequences are precisely the two which have not been included in any orthogroup (*Latimeria* BAHO01339439.1, *Rhincodon* LVEK03007534.1; Figure 2). However, according to synteny data, both are unrelated to *TNFSF-F6*. They may, respectively, be rapidly evolving *TNFSF-F1* (see genomic location in Figure 5; the gene is indicated as “TNFSF-other”) and *TNFSF15* genes (see below). Finally, for the EDA orthogroup, it is the presence of very similar genes in cyclostomes that diminishes the bootstrap values (Figure 2). However, the branch that includes both the gnathostome *EDA* genes and these cyclostome *EDA-like* genes has 100% bootstrap support (Figure 2), strongly suggesting that the cyclostome sequences may also be *EDA* orthologs, a result that will be examined in detail below. In any case, despite low bootstrap support values for these three proposed orthogroups, TNFSF15, TNFSF-F6, and EDA, synteny data indicate that the genes included in them are true orthologs, as they are located in the same regions in multiple species (Figure 6, Figure 7 and Figure 8).

Let us now consider in detail the orthogroups that are defined for the first time here, TNFSF-F6 to TNFSF-F9. As already indicated, the sequences of the genes included in each of these groups are very similar, so they appear together in the tree. Synteny provides additional useful information. For example, it indicates that *TNFSF-F6* is most likely an ancient *TNFSF-F1* tandem duplicate, present in both chondrichthyan and osteichthyan fishes (Figure 5). The chondrichthyan sequences actually correspond to those called “TNFSF33” by Redmond et al. [13], which, however, failed to detect the osteichthyan ones. *TNFSF-F7* and *TNFSF-F8* genes are both found adjacent to *TNFSF4* and *TNFSF6* (Figure 6). Despite this, it is very unlikely that they correspond to the same gene, given how different their sequences are (see positions in Figure 2) and the fact that they appear only in a few closely related species: *TNFSF-F7* in *Protopterus* and *Latimeria* and *TNFSF-F8* in chondrichthyans. The simplest explanation for the origin of these two genes is that they are independently emerging, slow-evolving duplicates of *TNFSF4*. The logic to reach that conclusion starts by considering that these three orthogroups, TNFSF4, TNFSF-F7, and TNFSF-F8, appear relatively close in the tree (Figure 2). Moreover, the region where they reside and the one where *TNFSF7* and *TNFSF9* genes are present (shown in Figure 9) have a common origin, emerging as two separated regions due to WGD2 (as first indicated by Collette et al. [6]). Therefore, the fact that *TNFSF4* genes are more distant in the tree from *TNFSF7* and *TNFSF9* than *TNFSF-F7* and *TNFSF-F8* can be explained by *TNFSF4* evolving at a faster rate than the latter two genes. Finally, the last new gene, *TNFSF-F9*, is found only in osteichthyans and is located in tandem with *TNFSF9* and *TNFSF7*, appearing quite close to those genes in the tree (Figure 9). The simplest hypothesis is that both *TNFSF7* (present only in mammals) and *TNFSF-F9* are duplicates of *TNFSF9*, which is much older, present in both chondrichthyans and osteichthyans (Figure 9).

As already indicated, putative shark *TNFSF3*, *TNFSF8*, and *TNFSF18* genes were described in Redmond et al. [13]. The presence of *TNFSF3* in sharks is confirmed here (Figure 5; Supplementary File S2). On the other hand, the other two associations were not supported by our data. The putative *TNFSF8* gene of *Rhincodon typus* described by Redmond et al. [13] was indeed detected. This corresponds to one of the two sequences already mentioned that could not be included in any orthogroup (LVEK03007534.1). However, as mentioned above, that gene is related to the TNFSF15 orthogroup. It appears relatively close to *TNFSF15* and radically distant from *TNFSF8* in our phylogenetic tree (Figure 2) and is located in tandem with *TNFSF15* in the *Rhincodon* genome (Figure 7). Thus, it can be interpreted as a shark-specific, relatively recent *TNFSF15* duplicate. Finally, their putative shark *TNFSF18* genes in fact correspond to those included here in the newly defined orthogroup TNFSF-F8. Significantly, neither in the trees obtained by Redmond et al. [13] nor in this study was a statistically significant association of *TNFSF18* and *TNFSF-F8* sequences detected. We can therefore conclude that putting together in a single orthogroup the *TNFSF18* and *TNFSF-F8* genes, as Redmond et al. [13] did, lacks support. Bona fide *TNFSF18* genes are found only in some tetrapod species.

The existence of the 24 gnathostome TNFSF orthogroups described in my first study [14] has been confirmed here. On the other hand, the addition of more species, not unexpectedly, has allowed for the characterization of four additional orthology groups. In contrast, the orthogroups called “GITRL (TNFSF18)”, “OX40L (TNFSF4)”, “4-1BBL/CD70(TNFSF9/TNFSF7)”, and “CD30L (TNFSF8)” by Redmond et al. [13] are not supported by these new data. It is important to note that they are lumped together in these putative orthogroups sequences that appear in particular branches of their tree, but support for three out of those four critical branches was statistically non-significant: the corresponding bootstrap values were just 93% (GITRL), 67% (OX40L), and 49% (4-1BBL/CD70). In “OX40L”, they grouped together genes belonging to the TNFSF4 and TNFSF-New orthogroups, an association that not only lacked bootstrap support, but also contradicted synteny data. As I already had shown [14], those two genes are in totally different locations, which in the human genome, respectively, correspond to 6p21 and 1q24. This indicates that Redmond et al. [13] had no evidence whatsoever to propose that orthogroup. Regarding the other three, when compared with my results, their “GITRL” group includes the *TNFSF18* and *TNFSF-F8* genes which I have discussed above, showing them to be different genes. In the “CD70 and 4-1BBL” group, they lumped together sequences that I have included here in orthogroups TNFSF9, TNFSF7, and TNFSF-F9. The presence of the three genes in *Danio rerio* (Figure 9) directly proves that they are paralogs and that three different orthogroups must be defined. For the fourth one (“CD30L/TNFSF8”), they obtained a significant value of bootstrap (97%) for the branch that includes *TNFSF8* with the chondrichthyan sequences. However, as I already described, that branch was not recovered in my analysis; the shark sequences appear relatively close to the *TNFSF15* genes, while bona fide *TNFSF8* genes (present only in sarcopterygians) are close to *TNFSF7* and *TNFSF9* and are very distant from *TNFSF15* (Figure 2). In conclusion, in all four cases, there was little to no evidence to propose those putative orthogroups. Significantly, these erroneous associations led Redmond et al. [13] to assume that the *TNFSF8* and *TNFSF18* genes were present in both chondrichthyans and osteichthyans, leading to an artificial inflation of the number of ancient genes in their model. The main reason for these discrepancies in the definitions of the gnathostome orthogroups are the radical differences in the topologies of the trees in Redmond et al. [13] and my two studies, which will be explained in the Section 4.

**Figure 10:**
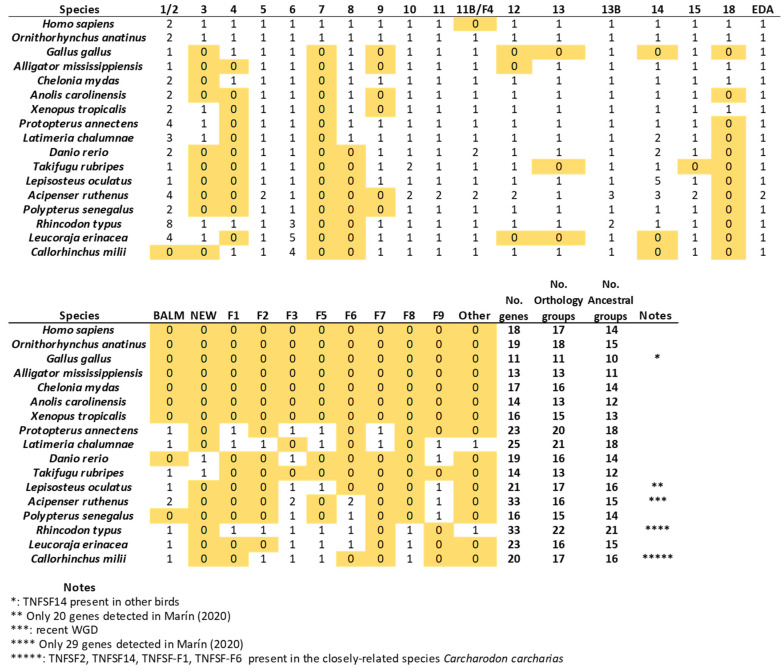
Gnathostome orthogroups. Number of genes belonging to each orthogroup in each model species is indicated. Top half: orthogroups present in tetrapods. Bottom half: absent in all analyzed tetrapods. The total number of genes, number of orthogroups, and number of ancestral groups, defined as those orthogroups present in both chondrichthyans and osteichthyans, are also specified for each species. Some genes were absent in these model species, but present in close relatives (see Notes).

In summary, 28 orthogroups have been identified, of which 21 are present in both chondrichthyans and osteichthyans, as summarized in Figure 10 (next page). Very significantly, these 21 ancient orthogroups are not only supported by sequence similarity (Figure 2), but also by synteny data, given that, for all of them, orthologous genes have been found adjacent to those of the TNF superfamily in at least a chondrichthyan and an osteichthyan species (Figure 3, Figure 4, Figure 5, Figure 6, Figure 7, Figure 8 and Figure 9; Supplementary File S3), demonstrating that the position of the genes today found in living species corresponds to the one in the ancestor of all gnathostomes. Therefore, twenty-one is the minimum number of genes present in that ancestor. Of course, additional genes, now lost in all living species, may have existed, but it is more parsimonious to assume that they did not if all data can be explained without recurring to them, which, as we will see, is the case here.

A final technical point concerns the fact that sampling additional sarcopterygian species in this study compared to my previous work [14] has indicated that genes in one of the orthogroups, which in that work were found only in fish species and thus called *TNFSF-Fish4*, are actually absent in eutherian mammals but present in the other tetrapod taxa (Figure 10). Given this result, it is preferable to give this gene a different name. Considering its high similarity to *TNFSF11* and its presence in some mammals, which allows to use the convention for mammalian genes, I propose naming it *TNFSF11B*. However, to allow an easier comparison between this study and my previous work, I will refer to it as *TNFSF11B/F4* throughout this text.

### 3.3. Deduction of Ancient TNFSF Genes Based on Tree Topology and Common Synteny

The second level of the dissection of the tree shown in Figure 2 concerns the information it provides regarding ancient genes from which the ones present in modern species are derived. In my previous work, I identified five clusters of closely related genes, which I named TNF, FASL, 1-1BBL, CD40, and EDA. I hypothesized that these clusters descended from five ancestral genes emerged before WGD2 [14]. It is simple to determine, comparing Figure 2 above with the trees presented in that work, that these five groups are again identified here. This was obviously expected, given the strong similarity among the genes within each cluster. However, there is a single significant difference in the deepest topology of the tree found here with respect to that obtained in my previous work, substantially altering how these five clusters must be interpreted. This difference concerns the position of the EDA cluster genes (*EDA*, *BALM*, *TNFSF13*, and *TNFSF13B*). These genes appeared in my previous work in a long branch, quite separate from the rest of the genes; their closest relatives, according to that topology, were *TNFSF-F2* (a TNF cluster gene) and *TNFSF6/TNFSF14*, the only two genes in the FASL cluster (see Figures 1 and 2 in [14]). However, in this new analysis, the EDA cluster genes appear in a totally different position, close to *TNFSF4* (Figure 2). This change, no doubt due to the improvement associated with the use of a better alignment algorithm, has a very important implication, which is that the topology found here perfectly agrees with how the genes are distributed across four different gnathostome chromosomes, something that was not the case with the topology obtained in my previous study. This observation allows for us to develop a very simple model of the evolution of the whole TNF superfamily. In Figure 2, four groups of genes, V11, V12, V21, and V22 (V meaning vertebrate), are indicated. The hypothesis that I will develop is that each of these groups descends from a single ancestral gene, which may be named *TNFSF-V11* to *-V22*, already present before WGD1.

Let us consider the data in detail. V11 genes correspond to those included in my previous work in the TNF and FASL clusters. In humans, they are found on chromosomes 1, 6, 9, and 19 in well-characterized regions, containing the MHC and its paralogons, known to be related, coming from a single region present before WGD1 [41]. Thus, it is reasonable to hypothesize that all these TNF and FASL cluster genes originated from a single ancestral gene, *TNFSF-V11*. There are eight different V21 genes: the ancient *TNFSF4* and *TNFSF9*, present in both chondrichthyans and osteichthyans, *TNFSF7* and *TNFSF18*, present only in tetrapods, and *TNFSF-F5*, *-F7*, *-F8*, and *-F9*, found only in fishes (details in Figure 10). *TNFSF4*, *TNFSF7*, *TNFSF9*, and *TNFSF18* were included in the 4-1BBL cluster, while *TNFSF-F5* was thought to be a TNF cluster gene with an unusually fast evolutionary rate, causing its abnormal position in the tree [14]. However, a simpler explanation exists for all these genes appearing together in Figure 2, which is that they all descend from a single progenitor gene, *TNFSF-V21*, present before WGD1. These genes are again found in humans on chromosomes 1, 9, and 19, in perfect agreement with what is observed for V11 genes, except that no V21 gene exists on chromosome 6, which may be explained by an early gene loss. Moreover, they not only appear in the same three chromosomes, but also are found in tandem with V11 genes in all three: *TNFSF-F5* is detected in tandem with *TNFSF15*; *TNFSF4*, *TNFSF18*, *TNFSF-F7*, and *TNFSF-F8* form a large tandem with *TNFSF6*; and *TNFSF7*, *TNFSF9*, and *TNFSF-F9* are together in another large tandem with *TNFSF14*. The simplest hypothesis to explain all these data is that the *TNFSF-V11* and *TNFSF-V21* genes were located in tandem before WGD1, and the tandem underwent two duplications, caused by WGD1 and WGD2, leading to the presence of related genes in four chromosomes of modern gnathostomes. Additionally, still more recent, duplications finally produced the full diversity of genes now detected in modern species on those chromosomes.

The exact same pattern emerges for V12 and V22 genes. V12 genes (*TNFSF5*, *TNFSF10*, *TNFSF11*, *TNFSF11B/F4*, *TNFSF12*, and *TNFSF-F3*) were, in my previous work, due to its strong similarity, all included in the CD40 cluster. They are located in regions that, in humans, correspond to chromosomes X, 3, 13, and 17, which also have a common origin, deriving from a single region present before WGD1 [15,16,21]. On the other hand, the four gnathostome V22 genes, which correspond to those ascribed to the EDA cluster, are found also on chromosomes X, 13, and 17 in humans. Moreover, as described for V11 and V12 genes, V12 and V22 genes may also appear in tandem: *TNFSF12* and *TNFSF13* are adjacent in many species. Therefore, the simplest explanation is again that they all derived from two ancestral genes, let us call them *TNFSF-V12* and *TNFSF-V22*, present in tandem before WGD1 and quadruplicated due to WGD1 and WGD2. A few subsequent duplications, losses, and chromosomal inversions are sufficient to explain the observed pattern in modern species.

Now, if we examine the deepest branches of the tree (Figure 2), it becomes obvious why the change in the position of the EDA cluster genes is so crucial. V11 and V12 genes are grouped together, occupying half of the tree, while V21 and V22 genes form the other half. This is exactly the expected relationship if all V11 and V12 genes derive from a still older gene, let us name it *TNFSF-V1*, while V21 and V22 genes come from a second one, originally located adjacent to the first, which may be called *TNFSF-V2*. This general explanation therefore postulates that a systematic expansion of two original TNFSF genes, due to the two whole-genome duplications plus several gene-specific duplications and gene losses, resulted in the current set of genes found in all living gnathostomes. Simple and elegant as this new hypothesis is, it still remains to be developed in full detail, gene by gene, and tested against the cyclostome data, which, as previously mentioned, provides direct insights into the situation after WGD1 but before WGD2. We will show below that cyclostome TNFSF genes fit perfectly within this new paradigm. However, before analyzing cyclostome species, it is necessary to consider another aspect of the evolution of the TNF superfamily in gnathostomes, namely how its complexity has evolved in different jawed vertebrate lineages.

### 3.4. Complexity of the TNF Superfamily Throughout Gnathostome Evolution

Once all the orthogroups in gnathostomes have been defined, it is possible to trace the origin of each one and analyze their patterns of survival or loss in the different lineages. In Figure 10, above, the number of genes, the number of different orthogroups observed, and the number of ancestral orthogroups, defined as groups that predate the chondrichthyan/osteichthyan split, have been detailed for each species. As already shown [14], the number of genes is quite variable. Among the model species examined here, *Rhincodon typus* and *Acipenser ruthenus* have the largest sets, with 33 genes each, and *Gallus gallus*, the smallest, with only 11 genes. Most of the genes in the sturgeon *A. ruthenus* appear in pairs, due to a whole genome duplication which occurred over 200 million of years ago [42]. It is noteworthy that the ancestor of all teleost fishes, such as *Danio rerio* and *Takifugu rubripes*, also underwent a WGD about 270 million years ago according to the latest estimates [43], but these two species have a relatively low number of TNFSF genes (Figure 10), indicating that, contrary to what happened in the *Acipenser* lineage, most duplicates were subsequently lost.

**Figure 11:**
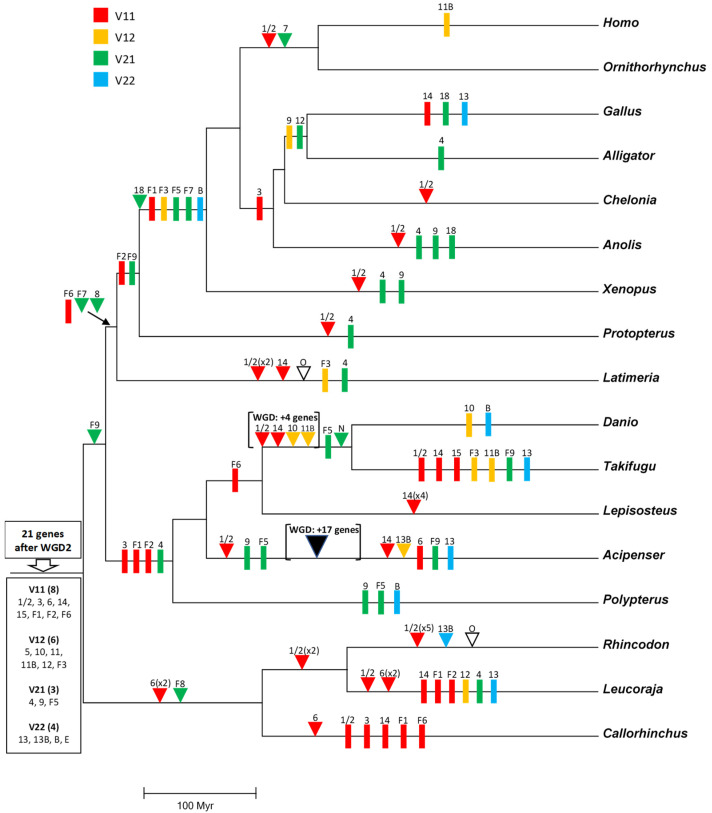
Species tree showing the most parsimonious explanation, in terms of gene duplications (triangles) and gene losses (rectangles), to explain the TNFSF gene sets found in the model gnathostomes. The tree topology and approximate divergence times were obtained from [44,45]. Colors indicate the ancestral genes (*TNFSF-V11* to *-V22*) from which each of the genes now observed in living species were derived. WGDs which occurred in the *Acipenser* lineage and also in the common ancestor of *Danio* and *Takifugu* are indicated. Genes named in the same branches but to the left of these WGDs appeared/disappeared before the WGD event, while those to the right reflect post-WGD events.

The number of orthogroups also varies widely across species (Figure 10). *Gallus* again has the smallest number (11), while the largest is also found in *Rhincodon typus* (22), although followed closely by *Latimeria chalumnae* (21) and *Protopterus annectens* (20). The notable difference between the number of genes and number of orthogroups in *R. typus* indicates that this species must have many duplicates of some genes. Indeed, Figure 10 indicates that eight genes belonging to the TNFSF1/2 group are found in *Rhincodon*, which largely explains this discrepancy. Finally, regarding ancestral orthogroups, present in both chondrichthyans and osteichthyans, 21 have been identified, as noted earlier. Strikingly, all of them are present in *Rhincodon* and most in *Latimeria* and *Protopterus* (18 in each species), but the rest of species have quite less, ranging from just 10 to 16. In my first study [14], I obtained data suggesting that, in the lineages leading to some modern gnathostome species (*Takifugu rubripes*, *Homo sapiens*), a significant reduction in the number of TNFSF genes occurred. Here, the analysis of many species allows for a more precise determination of the patterns of duplication and loss of TNFSF genes. Results are summarized in Figure 11 (next page), where genes have been color-coded according to the hypothesized ancestral genes (*TNFSF-V11* to *-V22*) from which they originated.

Figure 11 shows that it is not possible to find two model species with the same set of genes, indicating that the TNF superfamily has had a highly dynamic evolutionary history in gnathostomes. After the split of chondrichthyans and osteichthyans, a few genes emerged in each group, increasing the original number of 21 ancestral genes (Figure 11). Notably, three of them correspond to the newly described genes *TNFSF-F7* to *-F9. TNFSF-F6* is even older, existing already in the ancestor of all gnathostomes. Therefore, these four genes, absent in tetrapods, may have functions quite different from those described for mammalian TNFSF genes. Later, a trend towards the reduction in the TNF superfamily is observed in osteichthyans. Excluding lineages that underwent WGDs (such as *Danio*, *Takifugu*, and *Acipenser*), a total of 18 duplications and 32 losses are deduced in osteichthyan lineages. In contrast, in chondrichthyans, 16 duplications but only 11 losses are observed, and this number may be even lower, given that several losses were deduced to have occurred in *Callorhinchus milii*, but, in *Carcharodon carcharias*, a close relative of *C. milii*, there are genes of four orthogroups not found in the latter species (TNFSF1/2, TNFSF14, TNFSF-F1, and TNFSF-F6; see Figure 5 and Figure 9). These results suggest that some regions of the *C. milii* genome may remain uncharacterized, and those genes be present. The occurrence of multiple losses at certain branches is very interesting. A substantial reduction in genes occurred before tetrapod diversification (five genes lost and just one added), a trend that continued in saurian lineages. Before osteichthyan fish diversification, there were also four gene losses. Moreover, several lineages, such as those leading to *Gallus*, *Takifugu*, *Polypterus*, *Leucoraja*, and perhaps also *Callorhichus*, have undergone significant reductions in their TNF superfamily compared to their closest relatives (Figure 11).

Considering now the origin of the genes, notable differences are observed. V11 genes have a unique pattern, being duplicated more frequently than lost. This is largely due to the expansions of the TNFSF1/2 orthogroup genes in ten different lineages, in three cases involving multiple duplications, an extreme pattern not observed in any other orthogroup. Also contributing to the increase in V11 genes are two smaller expansions, which involve *TNFSF6* genes in chondrichthyans and *TNFSF14* in actinopterygian fishes. In contrast, V21 genes show a strong reductive trend, with only six duplications but twenty-one losses. On the other hand, for V12 and V22 genes, losses exceed duplications, but both are relatively rare. In summary, throughout gnathostome evolution the sets of V11 and V21 genes have changed very dynamically, while V12 and V22 genes have evolved more conservatively.

### 3.5. Characterization of Cyclostome Orthogroups and Relationships Between Cyclostome and Gnathostome Genes

In my initial study [14], I analyzed the TNFSF genes present in three cyclostome species, established cyclostome TNFSF orthogroups, and proposed a hypothesis linking each cyclostome orthogroup to a single gnathostome orthogroup. At the time, this one-to-one comparison was logical, as it was assumed that WGD2 occurred before the cyclostome/gnathostome split (see Section 1 Introduction). However, two important facts have since then emerged. First, as already mentioned, it is known that cyclostomes did not undergo WGD2; and, second, it has been established that a genome triplication occurred before the hyperoartia/myxini split, that is, affecting all cyclostomes, both hagfishes and lampreys [17,18]. Given this new evidence, a one-to-one comparison is no longer valid. In absence of gene losses, we would expect three similar but distinct cyclostome genes, generated by the cyclostome-specific triplication, to be equally related to two different gnathostome genes, emerged in WGD2. Of course, secondary gene duplications or losses could obscure this basic pattern.

Alongside the gnathostome orthogroups, Figure 2 indicates the positions of the cyclostome genes, which have been classified into six fundamental orthogroups, TNFSF-C1 to TNFSF-C6 (C = cyclostome), plus an additional one, TNFSF-C5b, which will be analyzed separately. This classification into just seven orthogroups is based on both sequence similarity and synteny data. For example, *TNFSF-C6* genes appear as three distinct branches, but synteny data confirm that they are orthologs; the division into three groups is driven by the combination of two factors, namely the presence of lineage-specific duplicates and the rapid sequence evolution of some genes. Comparing the tree in Figure 2 with the corresponding one in my initial study [14] reveals that six of the seven orthogroups, all but TNFSF-C5b, were already identified in my previous work. These six orthogroups appear in the same positions in both trees relative to the gnathostome genes. In summary, despite including three new cyclostome species and doubling the number of cyclostome genes analyzed, the results in both studies show nearly perfect congruence.

**Figure 12:**
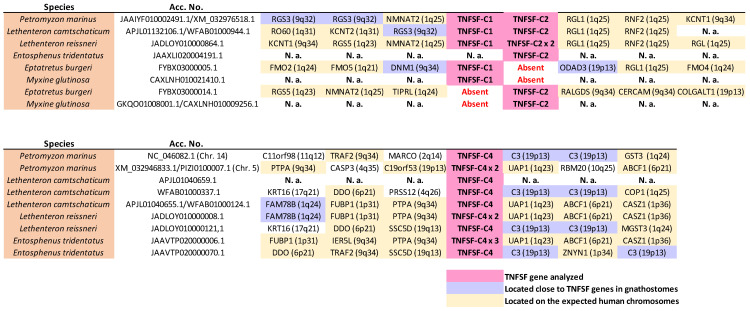
Synteny information for the regions surrounding the cyclostome genes *TNFSF-C1*, *-C2*, and *-C4* in lampreys and hagfishes. In this and subsequent figures containing synteny information, “N. a.” indicates that the corresponding region was not available in the databases.

**Figure 13:**
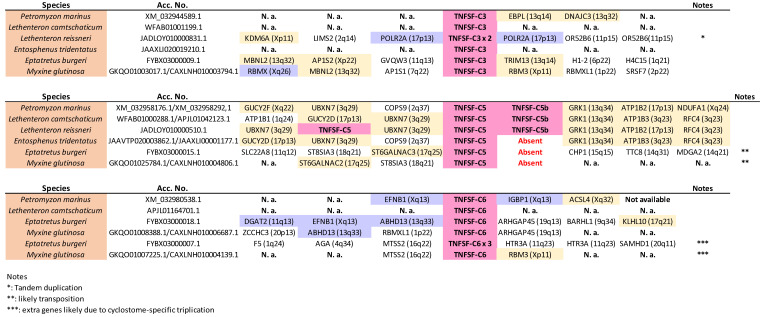
Synteny data for the regions where *TNFSF-C3*, *-C5*, *-C5b*, and *-C6* are located in cyclostomes. Color codes as in Figure 12. In this and subsequent figures containing synteny information, “N. a.” indicates that the corresponding region was not available in the databases.

The hypothesis developed in the previous section, which proposed that four genes, *TNFSF-V11* to *TNFSF-V22*, existed before WGD1, predicts that cyclostomes should possess four distinct classes of genes, positioned in specific positions of the phylogenetic trees. Figure 2 confirms this prediction: cyclostome genes are indeed found within each one of the regions of the trees hypothesized to derive from these ancestral genes. For instance, *TNFSF-C1* and *-C2* are located adjacent to the gnathostome genes deduced to come from *TNFSF-V11*, *TNFSF-C3* is located adjacent to gnathostome V12 genes, *TNFSF-C4* is close to gnathostome V21 genes and *TNFSF-C5*, and *-C6* is adjacent to the proposed V22 gnathostome genes. If these associations are due to shared, ancestral orthology, a second prediction is that the cyclostome TNFSF genes should be flanked by particular gene sets. Around V11 and V21 genes, one would expect to find genes whose orthologs in humans are located on chromosomes 1, 6, 9, and 19, while around V12 and V22 genes, the adjacent genes should have human orthologs on chromosomes 3, 13, 17, and X. This is confirmed in Figure 12 and Figure 13 (next page), which display synteny data for the regions containing cyclostome TNFSF genes. *TNFSF-C1* and *TNFSF-C2* are found in tandem in lampreys but separated in the only hagfish for which synteny data are available, *Eptatretus burgeri* (Figure 12). However, in all cases, they are surrounded by genes whose most likely orthologs in humans are on chromosomes 1, 9, and 19, as predicted. Moreover, two of those adjacent genes show strong similarity to *RGS3* and *DNM1*, which are adjacent to the V11 genes *TNFSF3* and *TNFSF15* in some gnathostomes (see Figure 5 and Figure 7). On the other hand, *TNFSF-C4* genes were detected in at least two different places in the genomes of lampreys (probably in three in *Lethenteron camtschaticum*, but data are inconclusive; they are not found in hagfishes), probably due to the cyclostome-specific triplication (see details in the next section). In one of those locations, one to three very similar duplicates in tandem are found, while in the other, single *TNFSF-C4* genes are observed (Figure 12). Again, as expected, most of the surrounding genes have their most likely human orthologs located on chromosomes 1, 6, 9, and 19. Moreover, one of them is very similar to the human gene *C3*, which is adjacent to the *TNFSF9* and *TNFSF14* genes in several gnathostomes (Figure 9). These results fully agree with the results of the tree, which suggested that *TNFSF-C4* genes descend from the ancestral *TNFSF-V21* gene. If we now turn our attention to the rest of cyclostome TNFSF genes, the situation is again as expected according to our hypothesis.

Most genes adjacent to TNFSF-C3, -C5, -C5b, and -C6 likely have human orthologs on chromosomes X, 3, 13, and 17 (Figure 13). Moreover, adjacent to the TNFSF-C3 genes, POLR2A and RBMX orthologs can likely be found, which are close in some gnathostomes to, respectively, TNFSF12 and TNFSF5, both TNFSF-V12-derived genes. This agrees with the close sequence similarity observed between TNFSF-C3 and gnathostome V12 genes (Figure 2). On the other hand, adjacent to TNFSF-C6 genes related to human EFNB1, IGBP1, DGAT2, and ABHD13 can be detected, placed in some gnathostomes close to the V22 genes EDA, BALM, or TNFSF13B (Figure 8, Supplementary File S3). Hence, all the available data indicate that TNFSF-C6 is a descendant of the ancestral TNFSF-V22 gene. We are finally left to consider the TNFSF-C5 and -C5b genes. The very strong similarity of TNFSF-C5 with gnathostome EDA (Figure 2), as well as its adjacency to genes whose human orthologs are most often found on chromosomes X, 3, 13, and 17, indicate that TNFSF-C5 also derives from TNFSF-V22. On the other hand, TNFSF-C5b, which is present only in lampreys in tandem with TNFSF-C5, is quite an anomaly. In principle, it could simply be a recent TNFSF-C5 duplicate. However, its sequence is more similar to gnathostome V21 genes than to V22 genes (Figure 2). My hypothesis to explain the origin of this gene is based on what I described above for the fast-evolving gnathostome gene TNFSF4. The regions shown at the interface between V21 and V22 genes in the tree in Figure 2 may be considered to be filled up with fast-evolving genes that “escape” from the positions where they should be found. For example, in that tree, gnathostome TNFSF4 should be adjacent to TNFSF-Fish7 and close to TNFSF7 and TNFSF9, as discussed in a previous section, but it is distant from them. Similarly, I propose that TNFSF-C5b is closely related to TNFSF-C5, but its fast pace of evolution has generated sequences that appear to be quite separated in the tree. This argument may be extended to other genes. It may provide an explanation of why the branches that include all V12 or all V22 genes have significant statistical support (97% and 99%, respectively), but V11 and V21 genes are not similarly grouped (Figure 2). If we look at the positions of the main V11 and V21 cyclostome genes (i.e., TNFSF-C4 in the first case and TNFSF-C5 and -C6 in the second) and their three respective closest gnathostome relatives, we see that the branches that include them, which may be defined as “central” in the sense that they approximately indicate the place in the tree where the ancestral genes TNFSF-V11 and TNFSF-V21, would be placed if their sequences were available, have relatively high bootstrap support (83% and 89%, respectively; Figure 2). However, the rest of gnathostome V11 and V21 genes, as well as cyclostome TNFSF-C5b, become distributed progressively away from these branches. This can be interpreted through most V11 and V21 genes having quite fast evolutionary rates, which leads to their relationships becoming obscured, the topology being somewhat altered, and the support values, even for the central branches, being lower than expected. The rapid changes observed in the sets of V11 and V21 genes present in different species (see previous section) and this fast pace of sequence evolution may certainly have related causes.

These findings refute two previous claims: (1) My inference that, in early cyclostome evolution, the number of TNFSF genes was drastically reduced [14] cannot be sustained. Instead, the results shown above indicate that most genes present before the cyclostome/gnathostome split have been conserved in cyclostomes. In the next section, I will develop the simplest model to explain the early cyclostome data, showing that a few early gene losses have been compensated by some duplications. (2) Contrary to the model proposed by Redmond et al. [13] which hypothesized as many as 15 TNFSF genes after WGD1, the information obtained from cyclostome genomes points to a much smaller number. In the next section, I will show that the available data support the presence of only eight genes at that time, corresponding to four pairs, derived from the four ancestral genes, *TNFSF-V11* to *TNFSF-V22*, as a result of WGD1.

### 3.6. A Model for TNFSF Evolution in All Vertebrate Lineages

**Figure 14:**
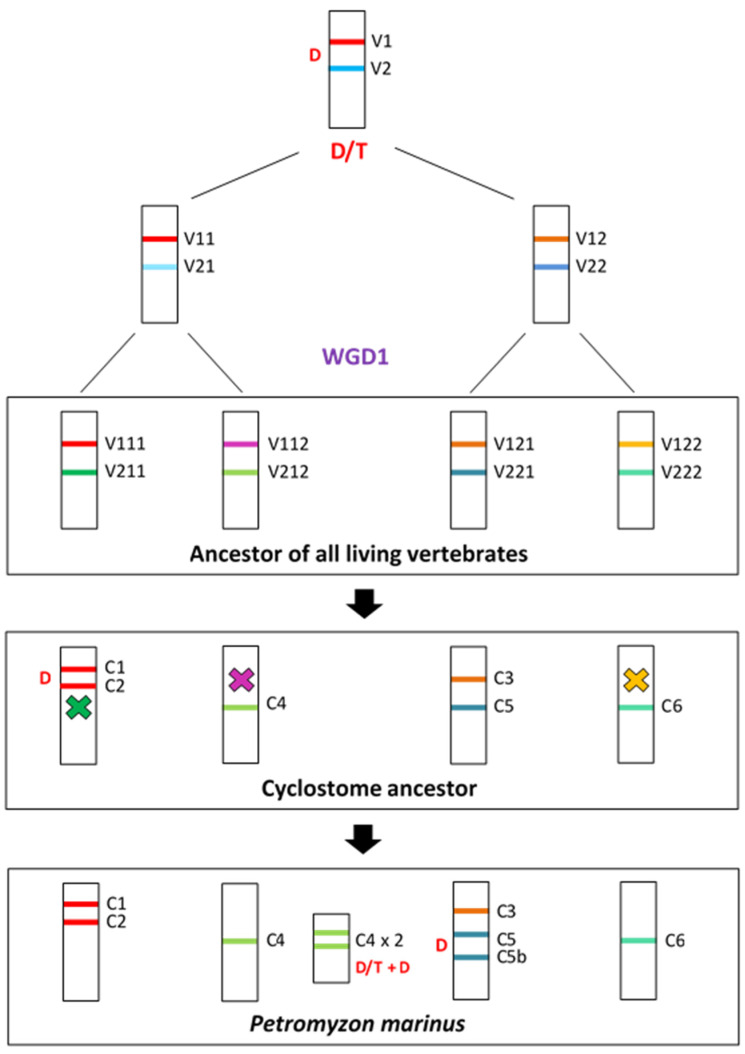
Early events in the evolution of the TNF superfamily in vertebrates and evolution in cyclostomes. The origin of the *TNFSF-V11* to *TNFSF-V22* genes is shown at the top. At the bottom, an example of the evolution of these genes in cyclostomes. D: duplication; D/T: duplication plus transposition. Crosses indicate gene losses. Colors are used to denote a common origin.

**Figure 15:**
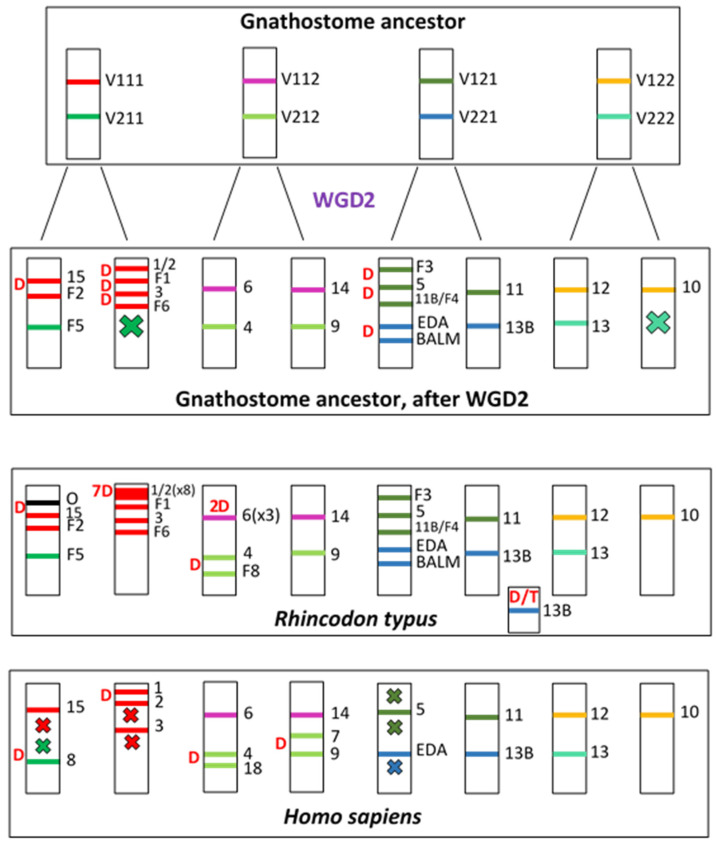
Evolution of the TNF superfamily in gnathostomes. *Rhincodon typus* and *Homo sapiens* are shown as examples of the remodeling of this superfamily since the origin of gnathostomes. From left to right, the human chromosomes are the following: 9, 6, 1, 19, X, 13, 17, and 3. Abbreviations as in Figure 14.

The general conclusion of the previous sections is that all the available data for both cyclostome and gnathostome TNFSF genes are compatible with the hypothesis of four ancestral genes existing very early in vertebrate evolution, before WGD1. This hypothesis is developed in full detail in the evolutionary model shown in Figure 14 and Figure 15 (see next pages). Figure 14 depicts the predicted genes in early vertebrate evolution and cyclostomes, and Figure 15 refers to gnathostome species. This model has two strong constraints: (1) it must account for all the available data from living species; and (2) it must be maximally parsimonious, minimizing the number of rare events, such as genome duplications, gene duplications, gene losses, transpositions of genes to new locations, etc.

The proposed evolutionary history begins with a single TNFSF gene which became duplicated in tandem very early in animal evolution. Later, this pair of genes duplicated again and transposed, resulting in four genes, precisely the ones which we have called *TNFSF-V11* to *-V22*, present in two different chromosomes (Figure 14, top). Comparing the current situation in living species with the available reconstructions of the ancestral vertebrate chromosomal set, these two chromosomes correspond to ancestral chromosomes 9 and 16 in Sacerdot et al. [21], CLGM and CLGN in Simakov et al. [15], or PVC15 and PVC6 in Nakatami et al. [16]. These four genes are the only ones which have to be hypothesized before WGD1 to explain all the other results. When WGD1 occurred, it led to a set of eight TNFSF genes in four different chromosomes in the common ancestor of all vertebrates. These genes may be called, following the same nomenclature, *TNFSF-V111* to *-V222* (Figure 14, center). To account for the genes present in cyclostomes, it is sufficient to postulate that three of those genes (*TNFSF-V211*, *-V112*, and *-V122*) were lost, while *TNFSF-V111* was duplicated in tandem. This led to a set of six genes, which correspond to the ones named above *TNFSF-C1* to *-C6*. That basic set of TNFSF genes has been slightly modified in different cyclostome lineages; due to additional tandem duplications, some gene losses plus the effect of the cyclostome-specific triplication occurred. For instance, in *Petromyzon marinus*, nine genes are found, the three additional ones corresponding to *TNFSF-C5b*, adjacent to *TNFSF-C5*, plus two additional *TNFSF-C4* genes (Figure 14, bottom). Two of the three *TNFSF-C4* genes are located in tandem on chromosome 5 of that species, while the third one is found on chromosome 15. This distribution in two different chromosomes in *Petromyzon* and other species (Figure 12) may be explained if *TNFSF-C4* genes became multiplied in the cyclostome-specific triplication, and some of them have been retained thereafter. The overall impact of that triplication on the set of cyclostome TNFSF genes has, however, been minimal, as no other TNFSF duplicates can be attributed to it (Figure 12 and Figure 13). This indicates that most TNFSF genes were lost following the genome triplication.

Figure 15 presents the simplest hypothesis to explain the evolution of TNFSF genes in gnathostomes. Following WGD1, four pairs of genes were present, each on a different chromosome (Figure 15, top). WGD2 doubled that number, resulting in 16 genes, which were later further amplified to generate the gene set present in the ancestor of all gnathostomes. It is deduced that seven duplications and two losses occurred since WGD1 that explain the final set of twenty-one genes in the gnathostome ancestor. Later, changes have been extensive, as summarized in Figure 11. Two particular examples have been shown on Figure 15 (bottom): first, *Rhincodon typus*, where, exceptionally, no gene has been lost since the ancestor of all gnathostomes and twelve duplications have occurred, largely increasing the total number of TNFSF genes; second, our species, in which seven losses have been partially compensated by four duplications, leading to the current set of eighteen TNFSF genes.

In summary, we can conclude that a quite simple model accounts for all the available data. This indicates that the evolution of the TNF superfamily in vertebrates has been largely determined by the impact of WGD1 and, in gnathostomes, WGD2, but not by the cyclostome genome triplication. Additionally, a considerable number of lineage-specific gene duplications and losses has led to a very variable set of genes, slightly different in each species. On the other hand, the models developed by Collette et al. [6] and Redmond et al. [13] cannot explain the available data. In the first case, the general outline is correct (four genes before WGD1, eight before WGD2), but their ancient genes only partially correspond to the ones deduced here. On the other hand, the Redmond et al. model is incompatible with the cyclostome data and also, as already demonstrated, contains some erroneous gnathostome orthogroups, which makes it also unable to explain some of the gnathostome results obtained here. Most significantly, their proposal of a very complex TNF superfamily already before WGD1 is untenable. On the other hand, my previous model [14] is quite similar to the one developed here, except in two particular aspects. First, my former model is less parsimonious, requiring some assumptions about the rates of evolution and the independent, asynchronous emergence of genes, which the current model avoids due to the new interpretation of the relationships of the genes of the EDA cluster with the rest of TNFSF genes and also the new identification of *TNFSF-F5* as a V21 gene. Second, as already indicated, the discovery of new orthogroups that contain genes present in both chondrichthyan and osteichthyan imply that the ancestor of gnathostomes possessed a somewhat more complex set of TNFSF genes than previously suggested. While I previously estimated 19 genes, current data indicate at least 21, which correspond to the ones that I then proposed plus *TNFSF3* and *TNFSF-F6*. It is formally possible but unlikely that this number will increase when more species are sampled, given that this work has explored all main gnathostome groups and also because Redmond et al. [13] examined four additional shark species without finding other candidates.

### 3.7. Functional Implications of the Evolutionary Data

**Figure 16:**
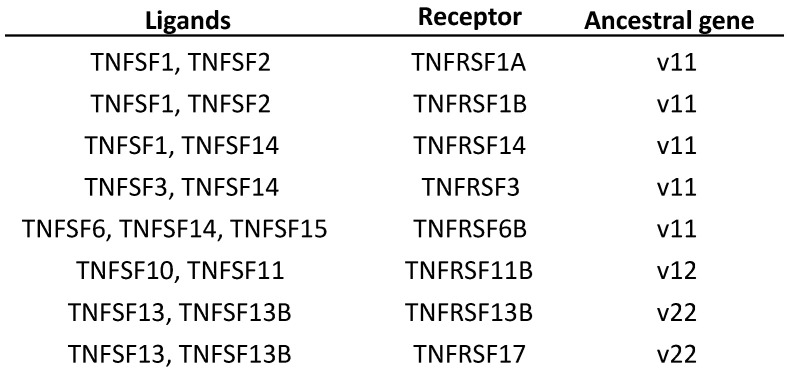
Genes encoding ligands which bind to the same receptors share a common ancestral origin.

A solid knowledge of the evolution of a gene family allows for a better understanding of its functions. Based on the model discussed above, several aspects of the functions of the TNFSF genes acquire a new meaning. One example is the fact that different TNFSF genes encode proteins which are able to bind to the same receptors of the TNFR superfamily. Sequence similarity alone does not fully explain some of these promiscuous interactions, given that some TNFSF proteins that share receptors are distantly related. For instance, TNFSF6, TNFSF14, and TNFSF15 products can all bind the decoy receptor TNFRSF6B/DcR3 [5] and, although *TNFSF6* and *TNFSF14* are closely related, *TNFSF15* is not (Figure 2). However, knowing the origin of these genes helps make sense of these observations. It turns out that all the TNFSF ligands that share receptors are derived from the same ancestral genes (Figure 16). Thus, although independent cooptions of unrelated ligands to interact with the same receptor cannot be ruled out, a more straightforward explanation is that, along the evolution of the superfamily, there have been episodes, e.g., after the WGDs, where the products of multiple TNFSF genes were able to interact with related receptors encoded also by different genes, many of them generated also in those WGDs, and that such an ambiguous situation has still not been completely sorted out.

Another example illustrating how a sound evolutionary model may illuminate functional aspects refers to the fact that there are four different ligands, the TNFSF4, TNFSF7, TNFSF9, and TNFSF18 proteins, which are expressed in dendritic cells and contribute to the priming of CD8+ T cells [46]. This multiplicity is difficult to interpret unless we consider that those four ligands are encoded by V21 genes. It is reasonable to hypothesize that *TNFSF-V21* itself had related functions in some cells present in the common vertebrate ancestor, which have been retained by its descendant genes despite hundreds of millions of years of divergent evolution. The presence of both lymphocytes [47] and cells which express dendritic cell markers [48] in cyclostomes suggests that these interactions could exist in the common ancestor of all vertebrates. The alternative explanation, that four related but quite different ligands and their corresponding receptors have been independently coopted to perform the same function, is quite less likely. No doubt, many other fine details of the functions of TNF superfamily members would be better understood within the evolutionary framework developed here.

In my earlier work, I provided evidence indicating that the involvement of TNFSF members in the regulation of apoptosis, through interactions with TNF receptors containing death domains, as well as their role in the activation of the NF-KB pathway, potentially leading to cell survival and to inflammatory responses, is very ancient, probably predating vertebrate emergence. However, given that those conclusions were based on a model of TNFSF evolution which has been here modified, it is pertinent to reconsider these issues. As in my previous study [14], I will conduct a retrograde analysis, starting with the evidence available for mammals and assuming that, if two genes share both a common ancestor and a given function, it is more parsimonious to consider that the ancestral gene already performed that function rather than the alternative of the function evolving independently twice. Applying this concept to the interaction with TNF receptors, it turns out that some V11, V12, and V22 genes encode proteins which interact with death-domain-containing TNF superfamily receptors (data for interactions between ligands and receptors obtained from [5]). Those are, respectively, *TNFSF1*, *TNFSF2*, *TNFSF6*, and *TNFSF15* (V11 genes), *TNFSF10* (V12 gene), and *EDA* (V22 gene). The simplest explanation for these six interactions is that the activation of death-domain receptors by TNFSF proteins predates the divergence of all these genes, implying that the ancestral gene from which all vertebrate TNFSF genes derived already possessed this capability. Later, V21 genes would have secondarily lost that ability. Given that exactly the same argument applies to the interactions of TNFSF proteins with TNF superfamily receptors lacking a death domain, we can conclude that the products of the original TNFSF gene, from which all the ones present in vertebrates emerged, most likely had the possibility of interacting at the same time with both types of receptors, with and without death domains. This is not a far-fetched hypothesis, given that it still occurs today. In the six cases mentioned above in which a TNF superfamily member encodes ligands able to bind death-domain-containing receptors, products of these same genes are also able to bind to one or several TNF superfamily receptors lacking those domains [5]. The same ancient origin can be inferred for the activation of the NF-KB pathway. All mammalian V11 and V22 genes as well as *TNFSF11* and *TNFSF12* (V12 genes) plus *TNFSF4* and *TNFSF9* (V21 genes) are involved in such regulatory functions [5]. Thus, a role in NF-KB pathway activation can also be traced back to the ancestral gene from which all current vertebrate TNFSF genes evolved. This type of backward analysis has the obvious problem that it can be affected by functional convergence. However, in all these cases, it is extremely unlikely that the products of so many, very different, genes independently acquired the same functions.

**Figure 17:**
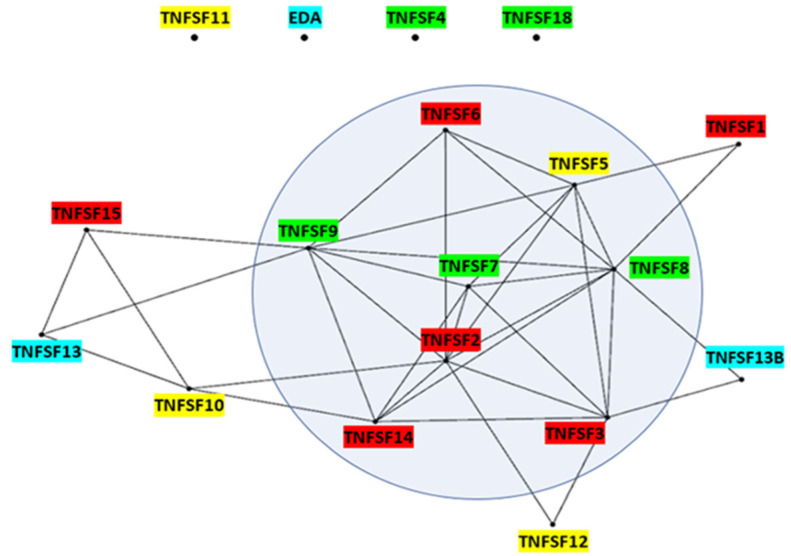
Network showing statistically significant correlations for TNFSF gene expression. Colors refer to the origin of the genes, as indicated in Figure 11 and the main text.

The patterns of expression of TNFSF genes may be examined in order to understand how the evolution of this superfamily relates with the development of the different systems in which it functions, particularly the immune system, in which TNFSF proteins have fundamental roles. Figure 17 shows the cases in which pairs of TNFSF genes have patterns of expression in 81 human cell types that are significantly correlated (Spearman’s correlation coefficient > 0.38; *p* < 0.05; see Section 2 Methods). A first interesting result is that there are four genes, *TNFSF4*, *TNFSF11*, *TNFSF18*, and *EDA*, for which no significant correlation was found. In the case of *EDA*, correlations were actually slightly negative for seven out of the seventeen comparisons. A second significant result is that eight genes, shown within a circle in Figure 17, are highly interconnected. Out of 28 possible positive correlations among them, 22 were found. The meaning of this group of genes becomes clear when the same analysis is repeated, but excluding the 11 cell types in the dataset which belong to the immune system. Then, only eight of those links persist, indicating that the correlations detected for these eight genes are largely due to similar patterns of expression in immune system cells. Finally, there are six genes which have only one or two links with the genes of the main group, so they appear in the periphery of the network (Figure 17).

Considering the origin of all these genes, it becomes obvious that V12 and V22 genes, shown in yellow and blue, respectively, in Figure 17, have fewer connections compared to V11 and V21 genes (in red and green, respectively). Genes belonging to the first two sets have, on average, only 2.6 links, while the latter two have a mean of 4.5 links. Also, only one V12 gene, *TNFSF5*, appears in the central core of highly interconnected genes, the other seven being V11 and V21 genes. This result suggests that the different dynamics observed along the evolution of gnathostomes (Figure 11) may be related to a different degree of involvement in immune functions. The emergence of new V11 and V21 genes, as well as the disappearance of some of them, could be due to their roles in rapidly evolving functions associated with the refinement of the system of adaptive immunity in gnathostome lineages. Conversely, V12 and V22 genes likely evolved more conservatively due to them often having more conventional functions in other systems. In this context, it is interesting to point out that, when knockout mice for these genes were generated, only two of them showed phenotypes which are unrelated with the immune system. They are *EDA* (a V22 gene) and *TNFSF11* (a V12 gene). *EDA* null mutants show very general anomalies, such as abnormal bones and skin, a lack of vibrissae and certain glands, among others [49,50,51,52]. *TNFSF11* mutants suffer severe osteopetrosis, tooth anomalies, and mammary gland defects [53,54]. Significantly, neither gene shows significant correlations with other TNFSF genes in our analysis (Figure 17).

**Figure 18:**
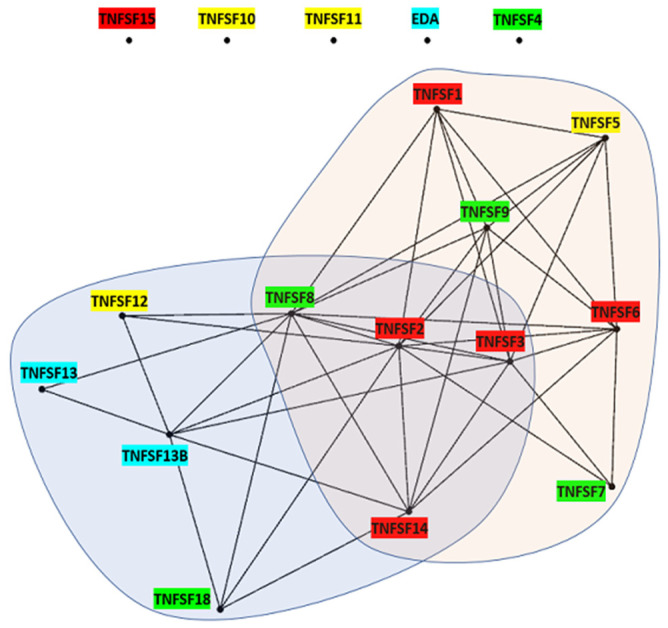
Network obtained when the expression of TNFSF genes in immune cells is analyzed. Blue cloud: genes highly expressed in macrophages and related cells. Pink: genes highly expressed in lymphocytes and NK cells. TNFSF2, TNFSF3, TNFSF8, and TNFSF14 exhibit both characteristics.

A second approach, focused on the patterns of expression in immune cells, allows us to refine the interpretation of the results just presented. Figure 18 (see next page) indicates the specific links among TNFSF genes when only immune system cells are considered. These links derive from a qualitative analysis in which two genes are considered similar if they have high levels of expression in three identical cell types out of the eleven immune system cells for which data were available. This type of analysis allows us to establish from which particular cell types the connections derive. In Figure 18, the network of TNFSF genes has been divided into two groups. Genes in the first group, encircled in blue in that figure, are connected due to them having high levels of expression in macrophages and related cells (such as monocytes, Langerhans cells, Hofbauer cells, etc.). These cell types can be considered to be part of the innate immune system. Cells similar in function to mammalian macrophages are very ancient, being present in invertebrates [55]. On the other hand, the connections of genes of the second group (enclosed in pink in Figure 18) are due to high expression levels in B and T lymphocytes and NK cells, closely related cell types of the lymphoid lineage, specific to vertebrate adaptive immunity. Included in both groups are four genes, *TNFSF2*, *TNFSF3*, *TNFSF8*, and *TNFSF14*, which have high expression levels in both macrophage-related and lymphocyte-related cells. This new analysis demonstrates that almost all TNFSF genes highly expressed in cells of the lymphoid lineage descend from either *TNFSF-V11* (red) or *TNFSF-V21* (green). Following with the idea developed in the previous paragraph, this may be interpreted as V11 and V21 genes having a central role in the refinement of the adaptive immune system, which may imply rapid changes, not only in their protein sequences or their functions but also in which genes remain critical and which ones become dispensable, when immune challenges are modified. The fact that the eight highly connected genes shown in Figure 17 are included in the set of genes connected by them having high levels of expression specifically in lymphoid cells (Figure 18) points to a deep relationship in the functions of all of them. It is also significant that two couples of genes in this set, *TNFSF1/TNFSF2* and *TNFSF7/TNFSF9*, derive from recent duplications (Figure 15), suggesting an advantage for increased complexity linked to refinements of the adaptive immune system in the mammalian lineage.

## 4. Discussion

The evolutionary dynamics of the TNF superfamily are complex. Here, I propose a novel model for the evolution of this superfamily, based on the broadest spectrum of vertebrate species analyzed to date. The conclusions largely support most of my previous findings [14], but at the same time refute a few aspects of that work. On the positive side, all the orthogroups identified in my previous study have been confirmed here, with only a few additional ones discovered in species not being included in my initial analysis. Also, the general model for the evolution of the TNF superfamily that I deduced then is similar to the one obtained here. However, the evolutionary history depicted in Figure 14 and Figure 15 presents clear improvements over my earlier version. In particular, it is simpler. It does not require three independent duplications before WGD1, but only two. Also, a specific gene, *TNFSF-F5*, which was hypothesized to be a TNF cluster gene—or, following the nomenclature used here, a descendant of *TNFSF-V11*—but with an unusually high rate of evolution, is now easily accommodated without resorting to such an ad hoc explanation: the new topology of the tree allows *TNFSF-F5* to be interpreted as a typical V21 gene. Another aspect of the simplicity of the model is its symmetry. From the ancestral pre-vertebrate organism to the ancestor of all living vertebrates, genes were duplicated but never lost, progressing from one gene to two genes in tandem, then to four genes in two tandems, and eventually to eight genes in four tandems, located in four different chromosomes, following WGD1 (Figure 14). This is the simplest possible sequence of duplications that could explain the current distribution of TNFSF genes in living vertebrates. It is satisfactory, after so much work, to find that it is perfectly compatible with all the available data. It is also noteworthy that greatly expanding the data, which even led to defining new orthogroups in both cyclostomes and gnathostomes, has actually simplified the evolutionary model for this superfamily.

On the negative side, certain limitations in my previous study have come to light. The most significant is unrelated to the quality of my original work and instead stems from the recent discovery that WGD2 occurred after the cyclostome/gnathostome split, and not before, as was assumed at the time of my earlier publication. This finding has led to two significant changes in the interpretation of the cyclostome data. First, in the past, I had to postulate a large number of losses in the cyclostome lineage following the cyclostome/gnathostome split. It is now evident that, immediately after this split, cyclostomes suffered only a limited reduction, from the eight genes present in the vertebrate ancestor to six in the common ancestor of all cyclostomes (Figure 14). The second significant change is that I proposed one-to-one relationships between cyclostome and gnathostome genes, which was logical if indeed both lineages had undergone WGD2, but incorrect if only gnathostomes did. Of course, the relationships become much more complex if WGD2 is gnathostome-specific, and, in parallel, cyclostomes underwent a specific triplication of their genomes. This study clarifies how the cyclostome and the gnathostome genes are related in the context of the general model proposed. Given the highly dynamic evolution of this superfamily, relationships are often intricate. For example, the cyclostome genes *TNFSF-C1* and *TNFSF-C2* are equally related to genes in six different gnathostome orthogroups. All of them were derived from the *TNFSF-V111* gene present in the common vertebrate ancestor (Figure 14 and Figure 15),

However, the idea that the TNF superfamily underwent, at some point, a significant simplification in cyclostomes may still hold. After their lineage-specific genome triplication, we would expect to find a very large TNF superfamily in all cyclostomes, perhaps even larger than that of gnathostomes, which only experienced WGD2. However, this is not the case. The number of genes attributable to the cyclostome triplication is minimal (see Section 3 Results). leading to the conclusion that the TNF superfamily suffered a dramatic decline following that event. This is a very interesting conclusion, which leads to the more general question of why TNFSF genes are conserved after some genome duplications but are lost relatively quickly after others. For instance, in early gnathostome evolution, the complete opposite of what happened in cyclostomes is observed: no gene losses occurred after WGD1 and only two have to be postulated after WGD2, the two losses being more than compensated for by seven independent duplications (Figure 15). Perhaps the simplest hypothesis to explain this striking difference between cyclostomes and gnathostomes is that the TNFSF genes were coopted for new tasks very early in gnathostome evolution to allow for the development of subtle systems of cell-to-cell communication in the context of an increasingly complex adaptive immune system [56,57]. In different circumstances, gnathostomes may also lose many TNFSF genes relatively soon after a WGD, as shown by the very limited number of genes conserved after the event occurred in the ancestor of *Danio* and *Takifugu*. As indicated in Figure 11, 13 of the 17 TNFSF gene duplicated in that WGD were lost in a relatively short period of time. Afterwards, even more genes were lost in the *Takifugu* lineage, although they have been conserved in *Danio* (Figure 11). On the other hand, the WGD occurred in the sturgeon lineage have almost duplicated the number of TNFSF genes, with gene losses occurring since being minimal at just three out of thirty-four genes (Figure 15). Thus, gene conservation is highly context-dependent, occurring only when duplicates can be functionally accommodated. The two main mechanisms for this are subfuntionalization, that is, partitioning the functions of each original gene among its duplicates or, alternatively, by the acquisition of novel functions in the context of increasing tissue or organismal complexity. The first process may explain the sturgeon case, given that the sequences of the *Acipenser* duplicates are strikingly similar, often nearly identical (see Supplementary File S3). The slow rate of rediploidization in sturgeons has been hypothesized to be related to their slow evolutionary rates at both the gene and chromosomal level [42,58]. The second process, the acquisition of new functions, was likely the main force for retaining so many TNFSF genes after the duplications occurred in early gnathostome evolution.

As already mentioned, the results obtained in this study refute the model proposed by Redmond et al. [13], which diverged from earlier models by hypothesizing a very complex TNF superfamily emerging very early in vertebrate evolution. Particularly, the cyclostome data cannot be interpreted within their framework without invoking numerous ad hoc gene duplications and losses, without any support in real data. However, it turns out that considering the cyclostome data is unnecessary to disprove their model. It is sufficient to critically analyze their study, which contains three fundamental errors. Among them, the minor one has been already mentioned above: in two cases, they put together unrelated chondrichthyan and osteichthyan genes without sufficient support for these associations, which artificially inflated the number of ancient genes that they had to postulate to fit the data. The other two problems are even more serious. One of them is that their model largely relies on the topology of the tree that they obtained; however, that tree was generated using a flawed methodology. From the outset, I was puzzled by the fact that their study and mine sampled species of the same groups and used basically the same methods of analysis, but resulted in radically different tree topologies. Thus, I decided to request the original alignment used in Redmond et al. [13] to verify that their analyses were reproducible. With that alignment, kindly provided by the corresponding author of the study, and using their exact methods, the ML tree obtained by those authors was duly recovered, with only very minor differences due to sampling effects. However, I noticed that their alignment included sequences ranging in length from 36 to 478 amino acids, while the only alignable portion in all TNFSF proteins, the THD domain (see Section 2 Methods), comprises approximately 150 amino acids. It is obvious that including sequences of very different sizes and non-conserved regions is going to haphazardly affect tree topology. This practice introduces spuriously aligned regions and artificially forces the alignment programs to generate an extremely large number of gaps, which may alter the topology in totally unpredictable ways (see e.g., [59]). The third, and most conspicuous, error is that they did not make any use of parsimony to build their model. The clearest example concerns the origin of the V12 and V22 genes found in extant vertebrates. My model demonstrates that all the data for those genes can be explained by the presence of just two ancestral genes, *TNFSF-V12* and *TNFSF-V22*, arranged in tandem on a particular chromosome prior to WGD1 (Figure 14 and Figure 15). However, Redmond et al. [13] postulated as many as six genes in that chromosome to explain the same results. This striking discrepancy stems from their failure to recognize that *EDA*, *BALM*, *TNFSF13*, and *TNFSF13B* may all come from a single gene and that the same may be hypothesized for *TNFSF5*, *TNFSF10*, *TNFSF11*, *TNFSF11B/F4*, *TNFSF12*, and *TNFSF-F3*. As shown in this study, all of them can be explained as emerging either in WGD2 or in subsequent gene duplications (Figure 15). The only reason for rejecting these possibilities, which, by the way, I had already proposed [14], is that they took the topology of their tree as an axiomatic truth. Let us, for example, consider the *EDA*, *BALM*, *TNFSF13*, *TNFSF13B*, and *TNFSF12* genes. The topology of the tree in Redmond et al. puts *TNFSF12* together with the other four, which, according to my analyses, is an artifact due to their faulty methodology. However, even so, they could have suggested that a single gene existed before WGD1 that gave rise to these five, with the *TNFSF12/TNFSF13* and *EDA/BALM* pairs emerging in two tandem duplications after WGD2. However, they surely discarded that simple hypothesis because in their tree, *TNFSF13B* and *BALM* clustered together while *EDA* and *TNFSF12* were separated from the rest. They assumed that topology to indicate the exact relationships among those genes, without other considerations—such as potential differences in evolutionary rates or the fact that *EDA/BALM* and *TNFSF12/TNFSF13* are found in tandem-no matter how that assumption was going to affect the complexity of their model. Consequently, they had to postulate a total of two duplications and five losses to explain a pattern that, in my models, ([14] and this study) requires exactly one duplication and zero losses. The five “ghost” genes, absent in all living species, which Redmond et al. [13] first created but later strategically made disappear, illustrate their departure from using criteria based on parsimony in favor of a literal acceptance of the (totally wrong) topology of their tree. Let us remember that disregarding parsimony allows us to postulate as many ancient TNFSF genes as we wish, given that it is always possible to concoct a model in which the fabricated genes conveniently disappear, latter in time, to fit the data.

We may ask now whether the model shown in this study is the final word regarding the evolution of the TNF superfamily. I believe that the basic features of the model, which are the steady increase in the number of TNFSF genes from the origin of vertebrates up to WGD1 (when the number of TNFSF genes was most probably eight), its slight early simplification in cyclostomes after the cyclostome/gnathostome split, the disappearance of many genes after the cyclostome-specific triplication, and the substantial increase in complexity in gnathostomes, due to the combination of WGD2 plus additional early duplications, but only very few gene losses (Figure 14 and Figure 15), are robust and unlikely to change. It is not by chance that I have finished up twice with very similar models, both in my first study [14] and here, despite significant differences in the species analyzed, the alignment methods, the topology of the trees obtained, or the quality of the available synteny data. Therefore, it would require finding species containing quite a large number of so far undiscovered ancient genes to shake these main conclusions, something that seems impossible considering the comprehensive taxon sampling in this study. However, further refinements, due either to improved analytical methodologies or more specific analysis, are possible. Particularly, some changes in the interpretation of the origin of certain V21 genes (e.g., *TNFSF18*, *TNFSF-C5b*), for which the topology of the tree and the synteny data are less definitive, are possible.

Having a solid model of the evolution of the TNF superfamily is very useful for interpreting functional studies. Above, I have shown several applications, such as explaining why some TNFSF proteins share receptors, deducing the types of TNF receptors that existed when vertebrates emerged, and the potential start of the TNFSF/TNFRSF control of the NF-KB pathway. Also, by analyzing in parallel the dynamics of the duplication and loss of TNFSF genes (Figure 11) and the patterns of expression of human genes (Figure 17 and Figure 18), I propose the hypothesis that V11 and V21 genes played the most critical role in the development of the gnathostome adaptive immune system, with V12 and V22 genes often having roles in other systems, as deduced, for instance, from the *EDA* and *TNFSF11* lack of correlation in expression with other TNFSF genes plus the phenotypes of the null mutants for those two genes in mice, or from the high levels of expression of several of these genes (*TNFSF12*, *TNFSF13*, *TNFSF13B*, and *TNFSF18*) in macrophages and related cells, but not in lymphocytes or NK cells (Figure 18), suggesting an early role of V12 and V22 TNFSF genes in innate immunity. The simplification of the TNF superfamily in a relatively short time in early tetrapod evolution (Figure 15) could also be related to significant changes in the adaptive immune system, such as an improvement in the mechanisms of humoral immunity to respond to antigen challenges (e.g., emergence of IgY in amphibians and, later, of the related IgG and IgE in other tetrapods; [60,61]), which may have allowed some cell-to-cell communication systems to become simplified. If this is correct, a prediction is that the analysis of the genes *TNFSF-F1*, *-F3*, *-F5*, *-F7*, and *BALM*, which were lost early in tetrapod evolution (Figure 11), could lead to the discovery of new functions or even perhaps new cell types in fishes, totally unrelated to the ones found so far in tetrapod species. These analyses and hypotheses of course far from exhaust the potential insights that understanding the TNF superfamily genes in an evolutionary context can provide.

## 5. Conclusions

The characterization of the TNF superfamily across all major vertebrate lineages has led to an updated model of its evolution, spanning from the last common vertebrate ancestor to all living species, both cyclostomes and gnathostomes. This model provides valuable insights into how the roles of TNFSF genes and proteins were acquired and how they have evolved in vertebrate species. It is hypothesized that rapid evolutionary changes in certain groups of evolutionarily related TNFSF genes correlate with their acquisition of new roles in the adaptive immune system.

## Figures and Tables

**Table 1:** Standard names of the gnathostome *TNFSF* genes, their equivalents in our species, and synonyms found in the literature.

Standard Name	Official Name *H. sapiens*	Other Aliases
***TNFSF1***	*LTA*	*LT*, *TNFB*, *TNLG1E*
***TNFSF2***	*TNF*	*DIF*, *TNFA*, *TNLG1F*, *TNF-alpha*
***TNFSF3***	*LTB*	*TNFC*, *TNLG1C*, *p33*
***TNFSF4***	*TNFSF4*	*OX40L*, *CD134L*, *CD252*, *GP34*, *TNLG2B*, *TXGP1*
***TNFSF5***	*CD40LG*	*CD40L*, *CD154*, *HIGM1*, *IGM*, *IMD3*, *T-BAM*, *TRAP*, *gp39*, *hCD40L*
***TNFSF6***	*FASLG*	*FASL*, *ALPS1B*, *APT1LG1*, *APTL*, *CD178*, *CD95L*, *TNLG1A*
***TNFSF7***	*CD70*	*CD27L*, *LPFS3*, *TNLG8A*
***TNFSF8***	*TNFSF8*	*CD30L*, *CD153*, *TNLG3A*
***TNFSF9***	*TNFSF9*	*4-1BB-L*, *CD137L*, *TNLG5A*
***TNFSF10***	*TNFSF10*	*TRAIL*, *APO2L*, *CD253*, *TANCR*, *TL2*, *TNLG6A*
***TNFSF11***	*TNFSF11*	*RANKL*, *TRANCE*, *CD254*, *ODF*, *OPGL*, *OPTB2*, *TNLG6B*, *hRANKL2*, *sOdf*
***TNFSF12***	*TNFSF12*	*TWEAK*, *APO3L*, *DR2LG*, *TNLG4A*
***TNFSF13***	*TNFSF13*	*APRIL*, *CD256*, *TALL2*, *TNLG7B*, *TRDL-1*, *UNQ383/PTRO715*, *ZTNF2*
***TNFSF13B***	*TNFSF13B*	*BAFF*, *BLYS*, *CD257*, *DTL*, *TALL-1*, *TALL1*, *THANK*, *TNFSF20*, *TNLG7A*, *ZTNF4*
***TNFSF14***	*TNFSF14*	*LIGHT*, *HVEML*, *CD258*, *LTg*
***TNFSF15***	*TNFSF15*	*VEGI*, *TL1*, *TL1A*, *TNLG1B*, *VEGI192A*
***TNFSF18***	*TNFSF18*	*AITRL*, *GITRL*, *TL6*, *TNLG2A*
***EDA***	*EDA*	*ECTD1*, *ED1*, *ED1-A1*, *EDA-A2*, *EDA1*, *EDA2*, *HED*, *HED1*, *ODT1*, *STHAGX1*

## Data Availability

All data required to repeat the analyses shown in this study are available either in the Supplementary Material Files or in the public databases cited in the text.

## Supplementary Materials

The following supporting information can be downloaded at https://www.mdpi.com/article/10.3390/biology14010054/s1: Supplementary Files S1–S3 (contents indicated in the main text).
